# Development, validation, and simplification of a scanner-specific CT simulator

**DOI:** 10.1002/mp.16679

**Published:** 2023-09-01

**Authors:** Sjoerd A. M. Tunissen, Luuk J. Oostveen, Nikita Moriakov, Jonas Teuwen, Koen Michielsen, Ewoud J. Smit, Ioannis Sechopoulos

**Affiliations:** 1 Department of Medical Imaging, Radboudumc, Nijmegen, The Netherlands; 2 Department of Radiation Oncology, Netherlands Cancer Institute, Amsterdam, The Netherlands; 3 AI for Oncology, Netherlands Cancer Institute, Amsterdam, The Netherlands; 4 Dutch Expert Centre for Screening (LRCB), Nijmegen, The Netherlands; 5 Technical Medicine Centre, University of Twente, Enschede, The Netherlands

**Keywords:** computer simulations, CT, system characterization

## Abstract

**Background:**

Simulated computed tomography (CT) images allow for knowledge of the underlying ground truth and for easy variation of imaging conditions, making them ideal for testing and optimization of new applications or algorithms. However, simulating all processes that affect CT images can result in simulations that are demanding in terms of processing time and computer memory. Therefore, it is of interest to determine how much the simulation can be simplified while still achieving realistic results.

**Purpose:**

To develop a scanner-specific CT simulation using physics-based simulations for the position-dependent effects and shift-invariant image corruption methods for the detector effects. And to investigate the impact on image realism of introducing simplifications in the simulation process that lead to faster and less memory-demanding simulations.

**Methods:**

To make the simulator realistic and scanner-specific, the spatial resolution and noise characteristics, and the exposure-to-detector output relationship of a clinical CT system were determined. The simulator includes a finite focal spot size, raytracing of the digital phantom, gantry rotation during projection acquisition, and finite detector element size. Previously published spectral models were used to model the spectrum for the given tube voltage. The integrated energy at each element of the detector was calculated using the Beer–Lambert law. The resulting angular projections were subsequently corrupted by the detector modulation transfer function (MTF), and by addition of noise according to the noise power spectrum (NPS) and signal mean-variance relationship, which were measured for different scanner settings. The simulated sinograms were reconstructed on the clinical CT system and compared to real CT images in terms of CT numbers, noise magnitude using the standard deviation, noise frequency content using the NPS, and spatial resolution using the MTF throughout the field of view (FOV). The CT numbers were validated using a multi-energy CT phantom, the noise magnitude and frequency were validated with a water phantom, and the spatial resolution was validated with a tungsten wire. These metrics were compared at multiple scanner settings, and locations in the FOV. Once validated, the simulation was simplified by reducing the level of subsampling of the focal spot area, rotation and of detector pixel size, and the changes in MTFs were analyzed.

**Results:**

The average relative errors for spatial resolution within and across image slices, noise magnitude, and noise frequency content within and across slices were 3.4%, 3.3%, 4.9%, 3.9%, and 6.2%, respectively. The average absolute difference in CT numbers was 10.2 HU and the maximum was 22.5 HU. The simulation simplification showed that all subsampling can be avoided, except for angular, while the error in frequency at 10% MTF would be maximum 16.3%.

**Conclusion:**

The simulation of a scanner-specific CT allows for the generation of realistic CT images by combining physics-based simulations for the position-dependent effects and image-corruption methods for the shift-invariant ones. Together with the available ground truth of the digital phantom, it results in a useful tool to perform quantitative analysis of reconstruction or post-processing algorithms. Some simulation simplifications allow for reduced time and computer power requirements with minimal loss of realism.

## 1 | INTRODUCTION

Currently, computed tomography (CT) is the workhorse imaging modality in most radiology departments.^1–4^ CT is used for screening, diagnosis, and interventional procedures, such as CT-guided biopsies or ablations.^5–9^ Therefore, research on reconstruction and post-processing algorithms to increase image quality in CT, without increasing patient dose, is a growing field of interest. Examples of these efforts include developments in deep learning reconstruction of low dose CT,^10^ in denoising of low dose CT using Convolution Neural Networks,^11,12^ and in CT denoising using statistical methods.^13,14^

New reconstruction or post processing algorithms are typically developed and validated using physical phantoms, which are limited in their capability to represent real human anatomy. This limits the usefulness of these phantoms when developing new algorithms. Patient images can also be used during development and validation, but aside from the ethical issues if new research-specific acquisitions are needed, patient images do not have a quantitative ground truth available, making it hard to quantify the performance of the developed algorithms.^15^

Therefore, it would be beneficial to have the possibility to test these algorithms using virtual clinical trials.^16^ In these, computer simulated images are generated from digital models of humans, such as the XCAT phantom.^17^ These phantoms have ground truth available and include considerably realistic anatomy, making them ideal for quantitative evaluation of clinically-relevant conditions. In addition, an infinite number of different realizations of the phantoms can be generated and infinite combinations of imaging conditions/parameters can be evaluated, thus the amount of data that can be used for a virtual clinical trial is only limited by computation time and memory.

However, simulating a fully detailed CT image that incorporates all acquisition process characteristics accurately is time and memory consuming. Therefore, for specific tasks it could be beneficial to evaluate the impact on realism of different simplifications that lead to substantially shorter computation time.

Therefore, we aim to develop a simulation of a scanner-specific CT capable of generating CT images with realistic appearance considering the spatial resolution and noise characteristics of a clinical CT system.^18,19^ With this scanner-specific CT simulation, a broad range of scanner parameters can be simulated, such as different tube currents, tube voltages, exposure times, bowtie filters, and focal spot sizes. In addition, once a full simulator is developed and validated, we aim to evaluate the impact on the realism of the resulting images when different simplifications (e.g., less subsampling) of the simulator are introduced, with the aim to reduce the time and computer power necessary to simulate an image.

## 2 | METHODS

**FIGURE 1:**
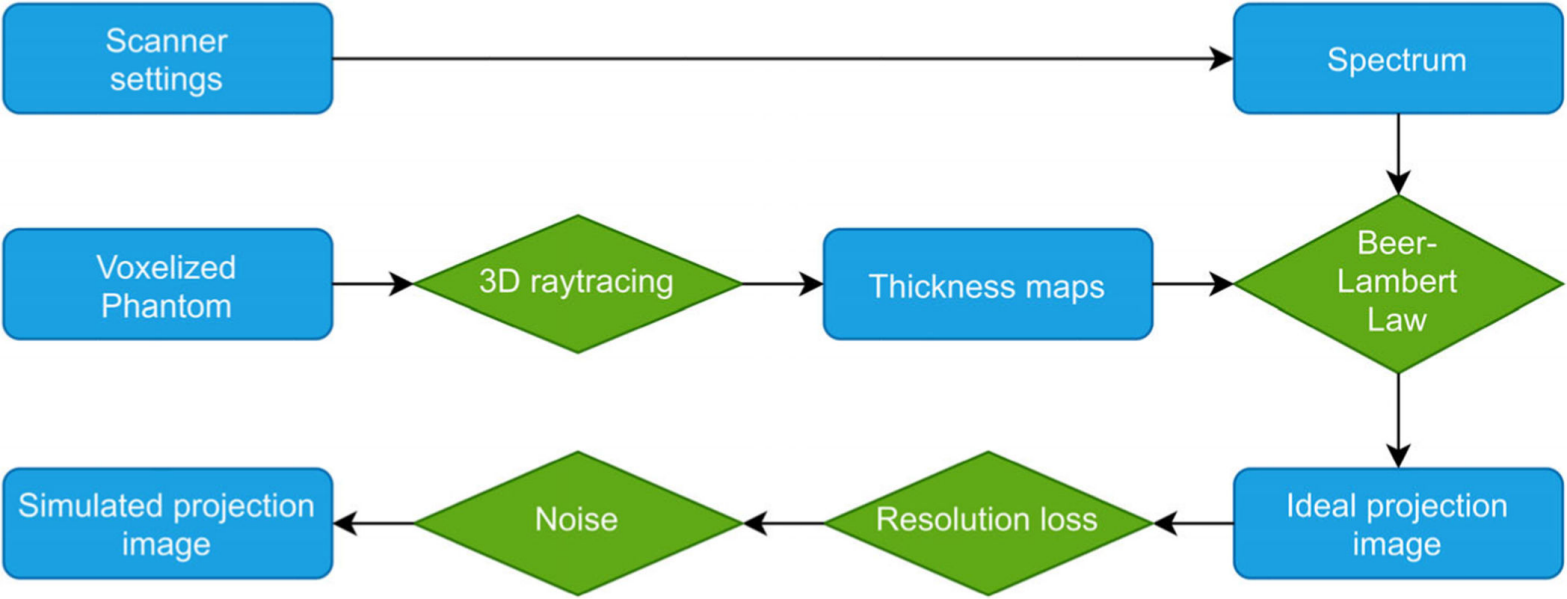
Scanner-specific computed tomography (CT) simulation pipeline.

The scanner to be simulated in this work is a 320-row CT system (Aquilion ONE PRISM Edition, Canon Medical Systems, Otawara, Japan) installed at the Dept. of Medical Imaging of Radboudumc, Nijmegen, The Netherlands. The scanner-specific CT simulation consists of a pipeline (Figure 1) to generate CT images that are realistic and scanner-specific, based on physics-based simulations for the position-dependent effects and image corruption methods proposed by Saunders et al. for shift-invariant corruption of ideal images.^20^ In this way the need for proprietary system-specific information from the vendor is minimized. However, for many of these steps, specific system characteristics need to be known, which, for this work, were obtained via measurements. The details of all steps and these measurements will be discussed in the next sections. A general simulation pipeline, with an overview of required information for each step, can be found in Appendix A (see online supplemental material).

### 2.1 | 3D raytracing

To perform the 3D raytracing, the specific geometry and dimensions of the clinical CT system, including the detector pixel size, detector distance, focal spot size, focal spot angle, and focal spot distance, were used. However, these are vendor-specific and confidential, so they are not reported here. For the CT system being simulated, the detector consists of 896 detector channels and 320 rows. The detector is curved such that all pixels in a row have the same distance to the source. The input to the 3D raytracing is a voxelized phantom, representing the object that will be imaged, with the voxel values indicating an index linking it to the material it contains. The 3D raytracing is performed for every material present in the phantom, resulting in a separate thickness map (T) for each material. The raytracing algorithm is a GPU-based pixel-driven raytracing based on the work of Moriakov et al.^21^ and Syben et al.^22^

**FIGURE 2:**
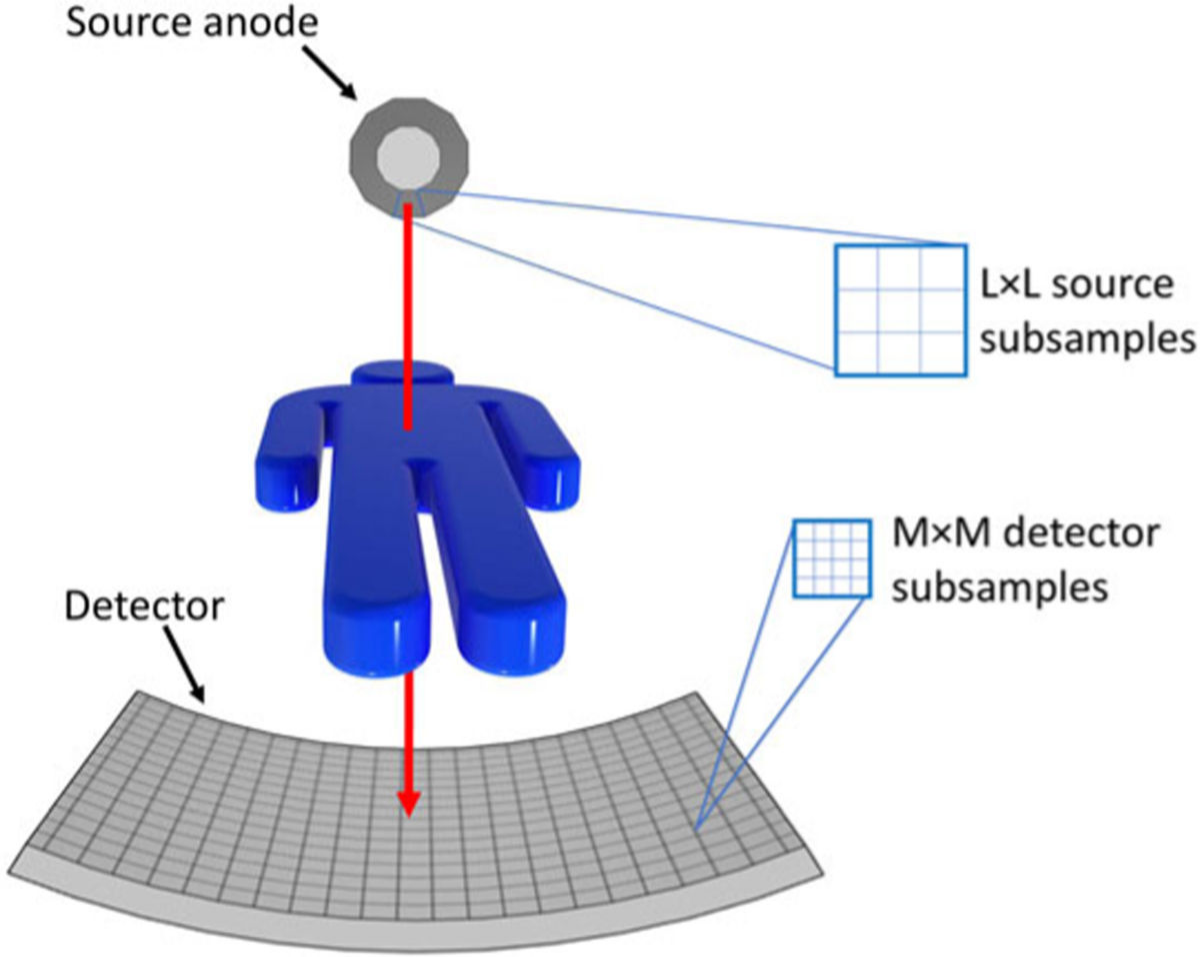
Schematic drawing of the focal spot and detector elements subsampling.

**FIGURE 3:**
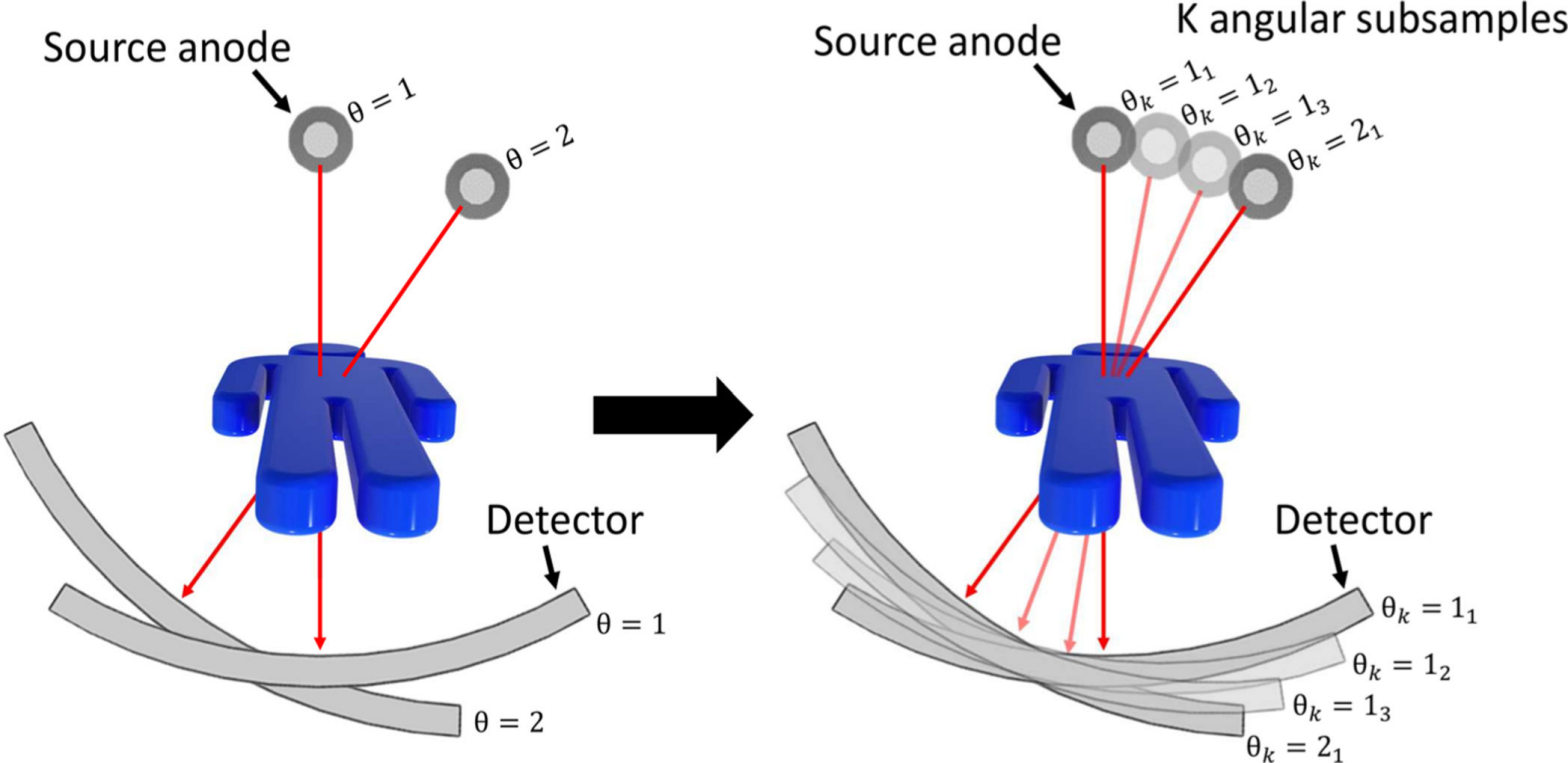
Schematic drawing of the angular subsampling.

To account for the finite size of the focal spot, the ray-tracing is not performed from a single point on the focal spot, but from *LxL* subsamples of the focal spot, ordered in a square grid, because the shape of the focal spot is approximately square. To minimize the discretization effect on the detector, the detector elements are subsampled by *MxM* accordingly and are up-sampled later in the simulation process.^20^ To incorporate the effect of the finite exposure time during the angular motion of the CT gantry that causes spatial resolution loss, the angular projections are also subsampled by a factor K. The focal spot and detector subsampling is depicted in Figure 2 and the angular subsampling is depicted in Figure 3.

This results in thickness maps containing the intersection length for each material from each focal spot subsample $f_{a,b}$ to the center of each subsampled detector element $\left(x_{i},y_{j}\right)$ at each subsampled angular projection $\theta_{k}$.

To include the effect of the bowtie filters in the simulation, the shape of the bowtie filters was determined using the method described by McKenney et al.^23^ For this, the air kerma was measured with a dosimeter (10 × 6–0.6CT, Radcal, Monrovia, California, USA) at the center of rotation (CoR) of the CT gantry and outwards in the lateral direction from the central ray, in steps of 5 mm out to 160 mm from the CoR, while the tube remained static. The resulting air kerma measurements were used to estimate the equivalent thickness of the bowtie filter.

### 2.2 | Primary projection images

To calculate the incident primary photon energy that is absorbed by the detector, first the x-ray spectrum (N) leaving the source must be determined. The x-ray spectrum is modeled by measuring the incident air kerma as close as possible to the detector (8 cm away) under four different attenuation conditions: no added attenuating materials, 6 mm aluminum, and 1 mm and 2 mm copper. These air kerma measurements are used to fit the spectrum model.^24^ The same measurements and attenuations are used to obtain the conversion from absorbed primary photon energy to digital units (DU). This conversion was determined for each bowtie filter separately.

As depicted in Figure 1, the x-ray spectrum is used together with the thickness map of each material to determine the primary photon energy absorbed by the detector, that is, the simulated sinogram $I\left(f_{a,b},x_{i},y_{j},\theta_{k}\right)$ according to the Beer–Lambert law^25^:

(1) $$I\left(f_{a,b},x_{i},y_{j},\theta_{k}\right)=\sum_{e}e*N_{e}*QE_{e}*\exp\left(-\sum_{m}\mu_{m,e}T_{m,f_{a},b,x_{i},y_{j},\theta_{k}}\right)$$

where $N_{e}$ is the number of photons with energy e emitted from the source, $QE_{e}$ is the quantum efficiency of the detector for each energy e, $\mu_{m,e}$ is the attenuation coefficient of material m at energy e (determined using the xraydb package in python based on the work of Elam et al.,^26^ and the work of Boone et al.^27^), and $T_{m,f_{a,b},x_{i},y_{j},\theta_{k}}$ is the thickness map of each material m at each subsampled detector element ($x_{i},y_{j}$) for each focal spot subsample $f_{a,b}$ and at each subsampled projection angle $\theta_{k}$.

### 2.3 | Spatial resolution loss

The spatial resolution characteristics of the detector are incorporated by applying the modulation transfer function (MTF) of the detector to the simulated sinogram resulting from Equation (1). This is done by multiplication, in frequency domain, of the MTF with the 2D fast Fourier transform (FFT) of the simulated sinogram (at each subsampled projection angle) and then taking the inverse FFT. Please note that the MTF needs to be divided by the sinc function of the final detector spacing, since the sampling of the detector causes the MTF to be multiplied by the sinc function.

The detector MTF was measured in the detector row direction, since this direction does not suffer from resolution loss due to rotation, and was used in all directions, assuming it is rotationally invariant. The slanted edge method^28^ was used with a tungsten edge (TX5, IBA Dosimetry, Schwarzenbruck, Germany). The tungsten edge was placed as close as possible to the detector (8 cm away) to minimize the focal spot size effect. A Lorentzian based fit^29^ is used to fit the MTF. The measured edge and fitted MTF are shown in Appendix B. The fit could potentially result in values close to the zero frequency to be larger than one. These are forced to one when applying the MTF.

The other causes of spatial resolution loss, namely, the focal spot size effect and the blur caused by exposure time per angular projection, are already included in the image, as described above, by raytracing the focal spot and angular projections, including subsampling. To maximize the realism of the image simulation, these effects must be included in the raytracing step, since they are position-dependent in the field of view (FOV), and therefore this information cannot be added to the sinogram directly.

After the incorporation of the detector MTF, the simulated sinogram is binned to its real dimension, using Equation (2):

(2) $$I(x,y,\theta )=\frac{1}{M^{2}*K*L^{2}}\sum_{i=1}^{M}\sum_{j=1}^{M}\sum_{k=1}^{K}\sum_{a=1}^{L}\sum_{b=1}^{L}I\left(f_{a,b},x_{i},y_{j},\theta_{k}\right)$$

where I(x,y,θ) is the sinogram after all subsamples are averaged at detector pixel x,y and angular projection θ, M is the number of detector pixel subsamples in each direction, K is the number of angular subsamples, L is the number of focal spot subsamples in each direction, $I\left(f_{a,b},x_{i},y_{j},\theta_{k}\right)$ is the sinogram with all subsamples, $f_{a,b}$ is the focal spot subsample, $x_{i}$, $y_{j}$ is the detector pixel subsample, and $\theta_{k}$ is the angular projection subsample.

### 2.4 | Noise addition

To add the correct noise to the sinogram two characteristics of the noise need to be known, the mean-variance relationship of the noise signal and the noise power spectrum (NPS). Both are determined using the same scans of two water phantoms of 240 mm and 320 mm in diameter, representing the attenuation of brain and abdomen, respectively.

The mean and variance of the signal were determined in a 20 × 30-pixel region of interest (ROI) at approximately the center of each sinogram projection, and their averages over all projections were used as the final mean and variance. To obtain the NPSs, first a correction for image lines due to detector tiling was performed by averaging all projections and subtracting the result from each individual projection. Second, the 2D FFT of a 64 × 64-pixel ROI in the center of the sinogram was calculated for each projection and the square of the absolute value of these FFTs was taken. The results were averaged for all projections, resulting in 2D NPSs. Despite the anisotropic pixel size there was no significant difference between the NPS in the horizontal and vertical directions, so they were radially averaged to obtain a 1D NPS. The mean-variance relationship is dependent on the tube voltage, and bowtie filter. The shape of the NPS is dependent on the tube current, tube voltage, exposure time, and bowtie filter. Hence, both were measured at nine different tube current levels between 10 and 400 mA, four different tube voltage levels, 80, 100, 120, and 135 kV, two different exposure times 0.275 and 0.5 s, and for two different bowtie filters.

The mean-variance relationship (*MV*) is defined as a linear function with a positive offset (Equation 3). This offset is the electronic noise.

(3) $$MV=a*m+b_{electronic\;noise}$$

where a is the slope of *MV*,m is the mean in a 3 × 3 pixel region (Saunders et al.^20^),and $b_{electronicnoise}$ is the offset due to the electronic noise.

To get the desired noise, white noise is generated with similar spatial dimensions as the sinogram projections, as described by Saunders et al.^20^ The resulting noise is multiplied in frequency domain with the square root of the NPS (Equation 4) and is scaled by the *MV* in spatial domain to obtain the desired noise, which is then added to the sinogram I(x,y,θ) (Equation 5).

(4) $$N(u,v)=\sqrt{NPS}*𝓕\{n(\mu =0,\sigma =1)\}$$

(5) $$I_{noise}(x,y,\theta )=I(x,y,\theta )+\sqrt{MV(I(x,y,\theta ))}\;*\left(\frac{1}{\sigma ({𝓕}^{-1}\left\{N\left(u,\;v\right)\right\}}*\left(({𝓕}^{-1}\left\{N\left(u,\;v\right)\right\}-\mu ({𝓕}^{-1}\left\{N\left(u,\;v\right)\right\})\right)\right)\;$$

where 𝓕 is the FFT operator, n is a realization of white Gaussian noise with mean μ and standard deviation σ, ${𝓕}^{-1}$ is the inverse FFT, N(u, v) is the colored noise image with the correct NPS $\sigma \left({𝓕}^{-1}\{N(u,v)\}\right)$ is the standard deviation and $\mu \left({𝓕}^{-1}\{N(u,v)\}\right)$ the mean of the colored noise in image domain after inverse FFT, *MV* is the mean-variance relationship, I is the primary projection image after the MTF is applied and binned and $I_{noise}$ is the projection image after the noise is added to it.

### 2.5 | Hounsfield unit calibration

As is standard in CT imaging, a linear calibration was obtained to apply to all reconstructed images to correct the resulting CT numbers for different materials and densities. The applied linear correction was determined by digitally simulating and physically measuring a cylindrical water phantom with 5 different inserts: Teflon, Delrin, acrylic, polypropylene, and air (quality control phantom provided by Canon Medical Systems) and fitting the CT numbers of the simulation to the physically measured CT numbers. The diameter of this phantom is 190 mm, and the inserts have a diameter of 20 mm. A linear correction was obtained from the mean HU of the simulated phantom inserts and water background and their corresponding theoretical values, using Equation (6).

(6) $$\underset{a,b_{water}\in \mathbb{R}}{\min}{\left(HU_{measurement}-a*\left(HU_{theoretical}+b_{water}\right)\right)}^{2}$$

The value of $b_{water}$ (offset of water) was fit such that the simulated water value corresponds to the theoretical one, that is, equal to zero. Afterwards a (slope) was fit such that the $HU_{theoretical}$, after correction, had the smallest possible error against the corresponding $HU_{theoretical}$. This was done separately for each tube voltage level available in the system, and the corresponding calibration was then applied to all subsequent simulated images.

### 2.6 | Validation of simulation

To assess the accuracy of the simulator, multiple validations were performed to validate the CT numbers of different materials, the spatial resolution, and the noise characteristics of the simulated images against images acquired with the clinical CT system. All validations were performed after reconstruction of the sinogram projections on the clinical CT system using the clinically available filtered back projection (FBP), which is based on the Feldkamp Davis Kress (FDK) algorithm.^30^ For the CT number and noise characteristics validation, the number of angular subsamples K was set to 2, the number of focal spot subsamples L was set to 1, and the number of detector subsamples M was set to 2. For the resolution loss validation, the number of angular subsamples K was set to 3, the number of focal spot subsamples L was set to 3, and the number of detector subsamples M was set to 4. These subsampling factors were obtained experimentally, the details can be found in Appendix C.

The CT numbers were validated using a physical oval phantom (with 40 and 30 cm radii for the horizontal and vertical directions, respectively) with 15 cylindrical inserts, each of different material and of diameter 28.5 mm^31^ (MECT phantom, Sun Nuclear, Middleton, WI, USA). The exact dimensions and material composition of the MECT phantom were known, so we could not only image but also simulate the phantom and its image acquisition, with a tube current of 400 mA and three different tube voltage levels (100,120,and 135 kV). The simulated voxel size of the phantom was 3.3 mm × 0.25 mm × 0.25 mm. The voxel size in the longitudinal direction was substantially larger since the phantom is constant in this direction. The measured and simulated sinograms were both reconstructed on the clinical CT system using FBP and a FOV of 320 mm × 320 mm and 160 mm in the longitudinal direction. The reconstructed volume consisted of 320 slices of 512 × 512 pixels. The hounsfield units (HU) within these inserts and in the water-equivalent background was measured by averaging a squared 10 × 10 pixel ROI across 80 slices.

The resolution loss of the simulator was validated by imaging a 50 *μ*m diameter tungsten wire,^32,33^ both digitally and physically. This tungsten wire creates a Dirac delta function or unit impulse,^34^ and the point-spread function (PSF) is obtained by taking the Radon transform^35^ of this signal in one direction. The MTF is then determined by calculating the FFT of the PSF. The spatial resolution was validated at 7, 14, and 21 cm from the isocenter, for both the digitally simulated wire and the real physical measured wire to verify the validity of the simulation of the shift-variant rotational blur and focal spot size effects. Each simulated and measured wire was reconstructed with a small FOV of 19.5 mm × 19.5 mm of 512 × 512 pixels, so the PSF had enough samples. The resolution loss was checked for both focal spot sizes present in the clinical system, which will be referred to as large and small focal spots from here on, and for both the radial and tangential direction for all positions. The simulated voxel size of the phantom was 0.1432 mm × 0.005 mm × 0.005 mm. The voxel size in the longitudinal direction was substantially larger and set to this exact value because with a shift of one pixel per longitudinal (the direction with pixel size 0.1432 mm) step this results in the simulated wire being placed at an angle of 3 degrees. Please note that the simulations were noiseless, since noise does not influence the resolution loss.

The resolution loss in longitudinal direction (across slices) has also been validated by imaging this 50 *μ*m diameter of the tungsten wire both digitally and physically. The wire was placed such that the angle with the slices was 8 degrees. The slice sensitivity profile (SSP) was determined in the same way as the MTF. The simulated voxel size of the phantom was 0.005 mm × 0.035 mm × 0.005 mm. The voxel size in the lateral direction was substantially larger and set to this exact value because with a shift of one pixel per lateral (the direction with pixel size 0.035 mm) step this results in the simulated wire being placed at an angle of 8 degrees. Please note that the simulations were noiseless, since noise does not influence the resolution loss.

The 50 *μ*m diameter of the tungsten wire is relatively small compared to the detector pixel size, even when subsampled. To overcome this problem the detector subsampling M was set to 24, just for the raytracing. After the raytracing, the detector was rebinned to its original subsampling of M=4.

For validating the noise magnitude and frequency content, a water phantom with a radius of 320 mm was again both digitally simulated and physically measured, and the results were compared. The noise magnitude and frequency content were validated at two different tube current levels (140 and 400 mA), and three different tube voltage levels (100, 120, and 135 kV). The simulated voxel size of the phantom was 1.0 mm × 0.25 mm × 0.25 mm. The voxel size in the longitudinal direction was substantially larger since the phantom is constant in this direction. A volume of interest (VOI) of 64 × 64 × 64 voxels was placed in the center of the water phantom images. The standard deviation of this VOI was used to validate the magnitude of the noise. To validate the noise frequency content, a 100 mm FOV was reconstructed in the center and at the periphery, approximately 120 mm from the center, of the water phantom. This smaller FOV was reconstructed, to have a smaller pixel size, making it possible to validate higher frequencies. The 2D NPS and 2D unstructured NPS of both these FOVs, were calculated in 256 × 256-pixel ROIs from across 80 slices, by determining the square of the 2D Fourier transform. In the case of the unstructured NPS the average of the 80 slices was subtracted before calculating the Fourier transform. Both the 2D NPS and 2D unstructured NPS were normalized to have an area of one, obtaining the normalized NPS (nNPS). Both the 2D nNPS and 2D unstructured nNPS were calculated to show that the simulation does not introduce any structured noise. A comparison of the nNPS at these two positions was performed to validate the changes in the noise characteristics throughout the imaging field. These 2D nNPSs at the center were also radially averaged, and again normalized to have an area of one, to obtain a 1D nNPS. To validate the frequency content across slices, the 1D nNPS was calculated across 280 slices for all pixels in a 128 × 128 ROI at the center, and the results were averaged.

Scatter was not included in our simulator, since the system performs scatter correction during the reconstruction process, and therefore, the benefit of adding simulated scatter would be minimal. To validate the performance of the scatter correction, the 320 mm water phantom (also used for the validation of the noise) was imaged with the standard volume scan collimation of 160 mm (equal to all measurements in this work) and with a 20 mm collimation, which is assumed to have a negligible amount of scatter. The line profile of the reconstructed water phantom images was compared for both collimations. Line profiles were obtained from these images by averaging 60 individual line profiles across 38 slices both horizontal and vertical directions for both water phantom scans and the corresponding simulation.

### 2.7 | Simulation simplifications

The three steps of the simulator incorporating subsampling, namely, the number of angular projection subsamples K, the number of detector subsamples *M* × *M* and the number of focal spot subsamples *L* × *L*, were simplified to reduce the time and computer power necessary. The angular subsamples *K* were set to 1, 2, and 3. The detector subsamples *M* × *M* were set to 1 × 1, 2 × 2, 3 × 3 and 4 × 4. The number of focal spot subsamples *L* × *L* was set to 1 × 1, 2 × 2, and 3 × 3. Please note that while one of these three was reduced the other two were kept at their original value. Previous CT simulators^18,36,37^ also used or optimized their subsampling, however with this analysis the impact of each individual simplification is shown.

To validate the impact of these simplifications on the realism of the simulation, the MTFs of the images resulting from the digitally simulated simplified sinograms were determined and compared to the MTFs of the physically measured sinograms. In both cases these MTFs were again determined from a tungsten wire at 7, 14, and 21 cm from the isocenter.

In addition, the possibility of compensating for simplifying the focal spot as being a point source by using the system MTF, that is, the MTF measured with the edge located at the CoR, instead of the detector MTF, was also tested. These MTFs are shown in Appendix B. Finally, the possibility of simplifying the incorporation of the rotational blurring, due to the angular motion of the source and detector, was also investigated by averaging each angular projection with the subsequent one, instead of performing the angular projection subsampling.

## 3 | RESULTS

**FIGURE 4:**
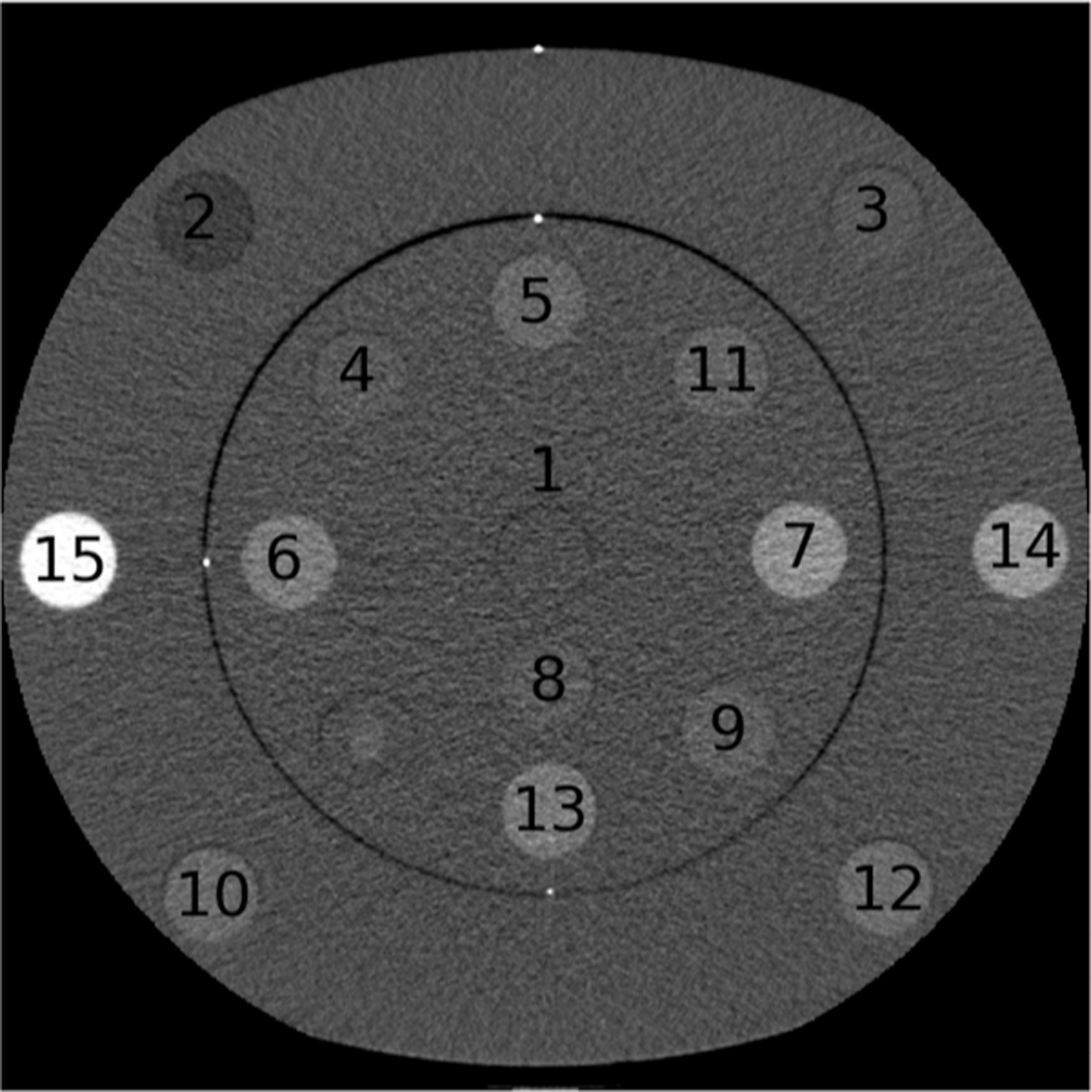
MECT phantom, measured with 135 kV, used for computed tomography (CT) number validation, with a window level (WL) of 200 HU and a window width (WW) of 1000 HU.

In Figure 4, one of the measurements of the MECT phantom used for the CT number validation is shown. Table 1 shows the measured and simulated CT numbers for the different materials in the MECT phantom, with the numbers corresponding to the regions in Figure 4. Table 1 also shows the maximum (bold and underlined), mean absolute, and mean error of the CT numbers in HU.As can be seen from the values in Table 1, the simulations result in a small negative bias in the CT numbers for all tube voltage levels.

**FIGURE 5:**
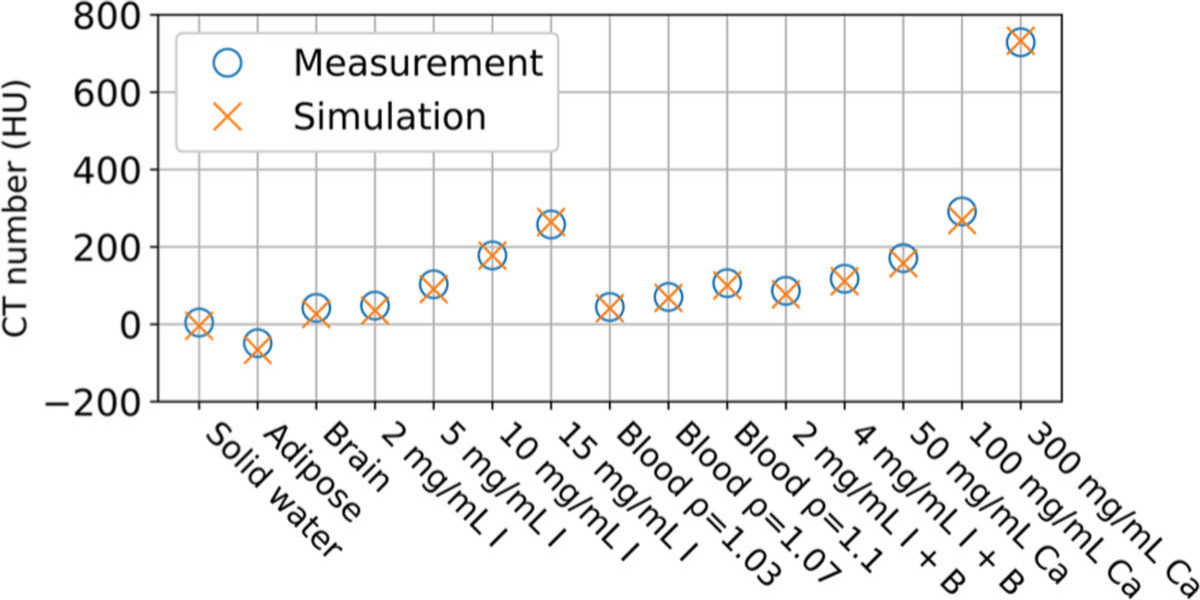
Computed tomography (CT) numbers of the real and simulated MECT phantom images at 135 kV. Note that the B stands for blood *ρ*= 1.03 g/cm.^3^

Figure 5 shows the measured and simulated CT numbers of the 135 kV case. The remaining errors in the CT numbers do not seem to have a correlation, indicating the simulation does not introduce any non-linear offset to the CT numbers. Note that, as explained before, the linear calibration is determined using a different phantom and is applied to all subsequent images.

**FIGURE 6:**
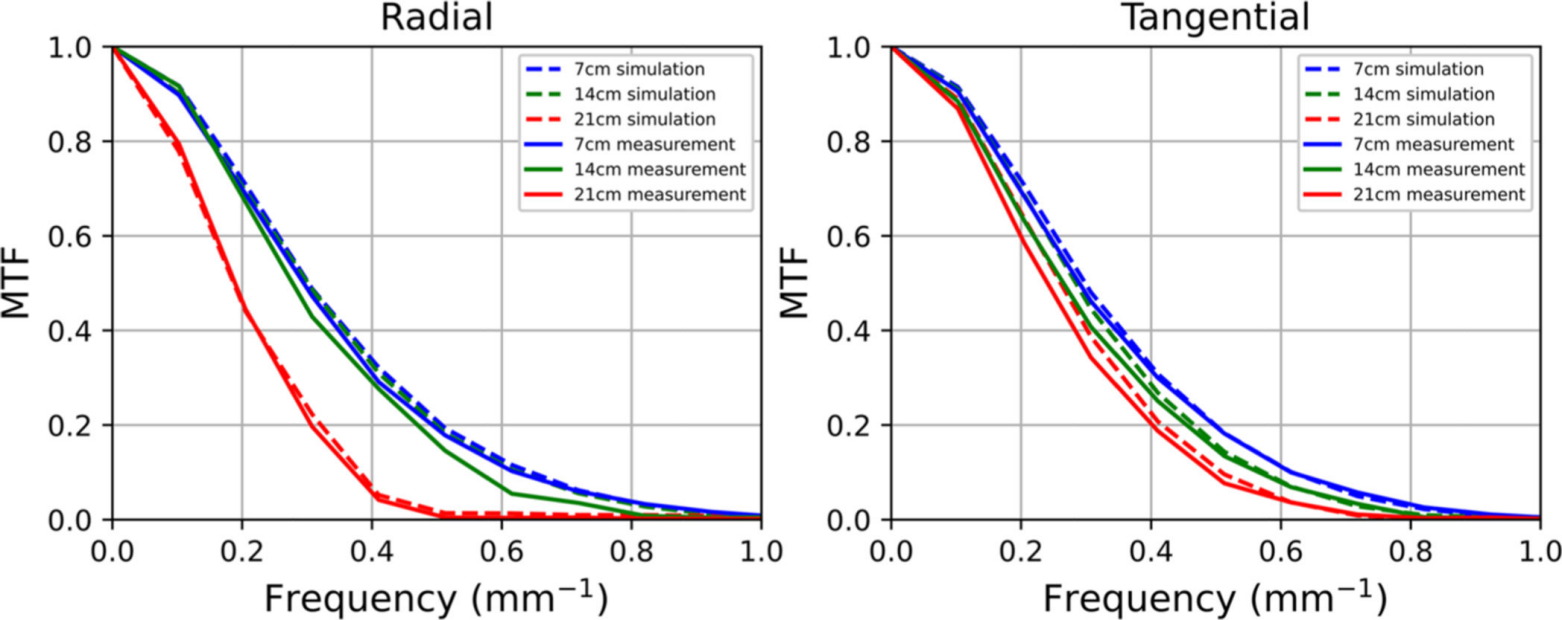
Modulation transfer function of measured and simulated wires in radial (left) and tangential (right) direction for the large focal spot.

The MTFs in the radial and tangential directions can be seen in Figure 6. It can be observed that the latter starts dropping when moving out of the CoR in both the measured and simulated cases. This is due to the rotation of the system introducing more blur further away from the CoR. The frequencies at 10% MTF and their relative error for the various FoV positions, directions, and focal spot sizes are listed in Table 2. The maximum error is 11.1% and the mean absolute error is 3.4%, showing that the spatial resolution characteristics in the simulated CT images are close to those of the clinical system.

**FIGURE 7:**
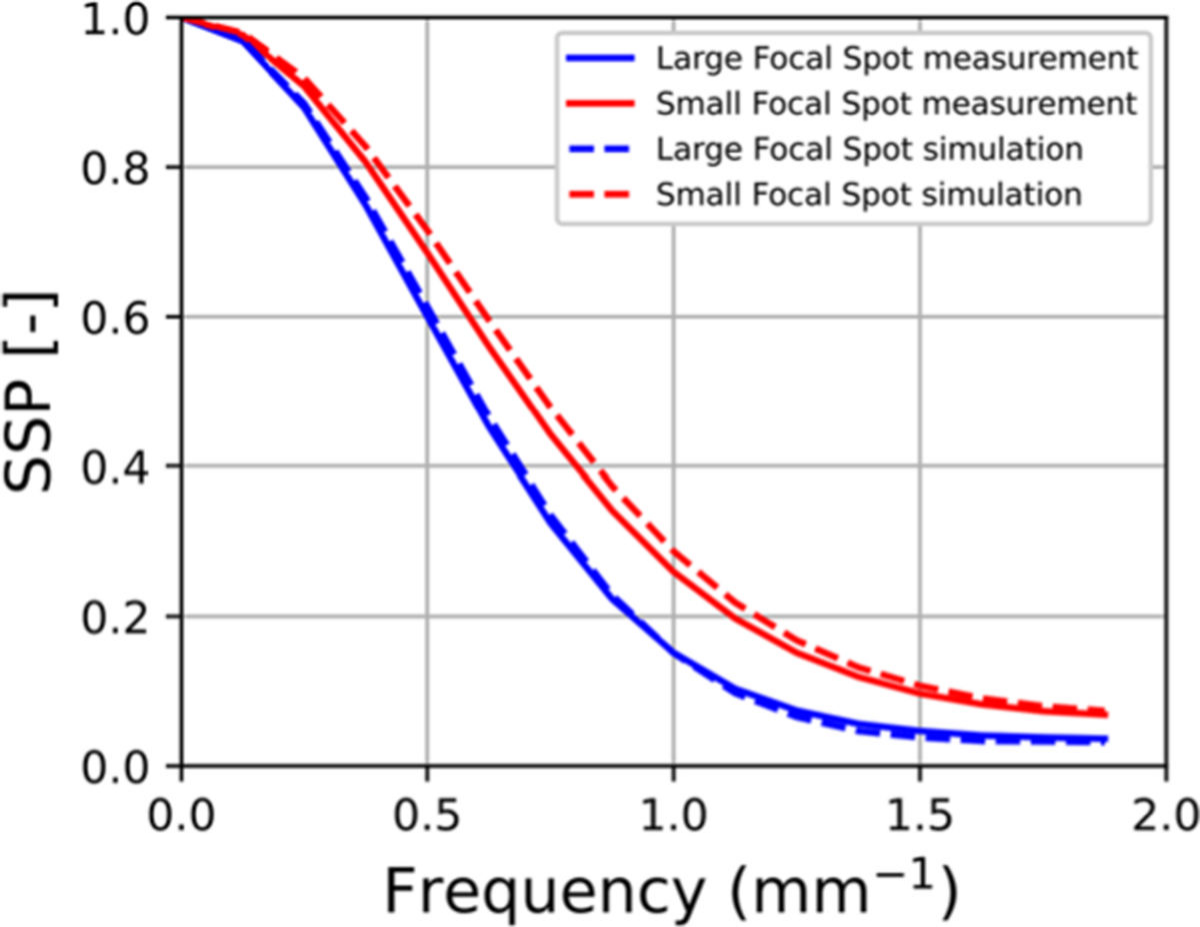
Slice sensitivity profile of measured and simulated wires for both focal spots present in the system.

The SSP of both the large and small focal spot can be seen in Figure 7. It can be observed that the resolution loss is higher for the large focal spot, as expected. Table 3 shows the frequencies at 10% SSP for measurement and simulation and their relative error for both focal spots. The mean absolute error is 3.3%,showing that the spatial resolution across slices in the simulated CT images is close to those of the clinical system.

**FIGURE 8:**
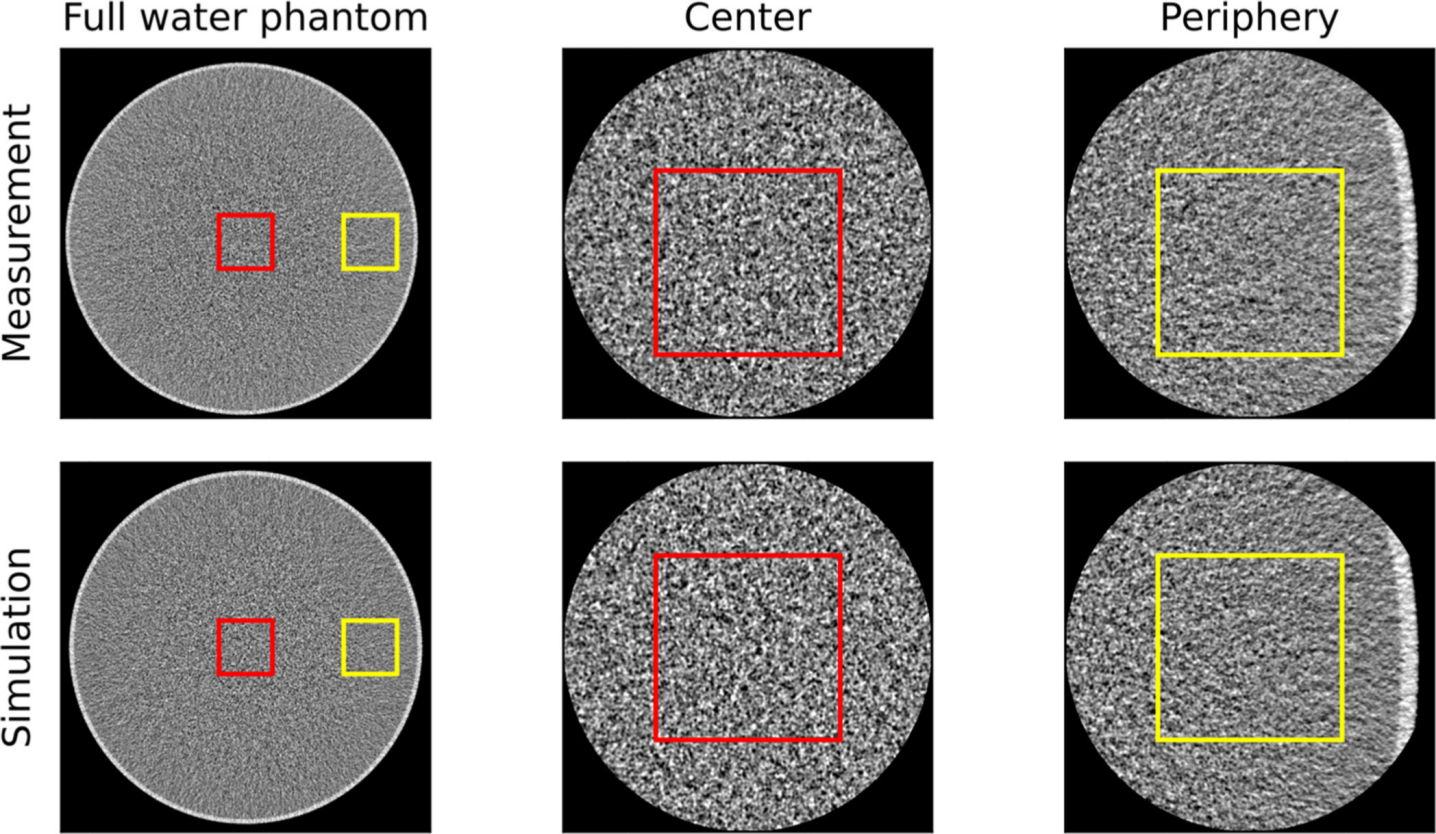
Water phantom used for nNPS validation (140 mA, 135 kV) with a WL of 0 HU and a WW of 400 HU. The squares indicate the ROIs used to determine the nNPSs.

Figure 8 shows images of the water phantom used to validate the noise characteristics of the simulated images. The red square indicates the ROI used for the validation of the noise magnitude. Table 4 shows the results of the noise magnitude in terms of standard deviation. The maximum and mean absolute errors were 8.5% and 4.9%.

**FIGURE 9:**
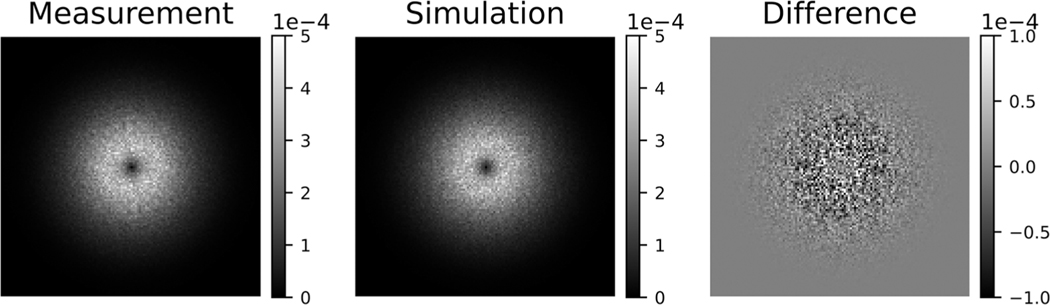
2D nNPS in the center region of measured (left) and simulated (middle) water phantom, and the difference between both nNPSs (right). Difference = simulation − measurement.

**FIGURE 10:**
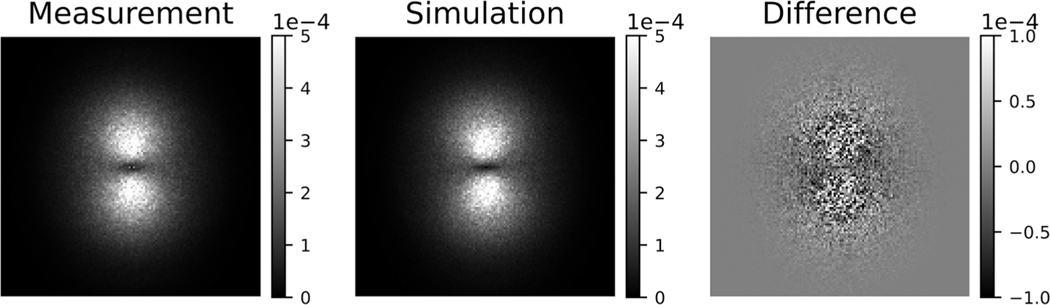
2D nNPS in the periphery region of measured (left) and simulated (middle) water phantom, and the difference between both nNPSs (right). Difference = simulation − measurement.

**FIGURE 11:**
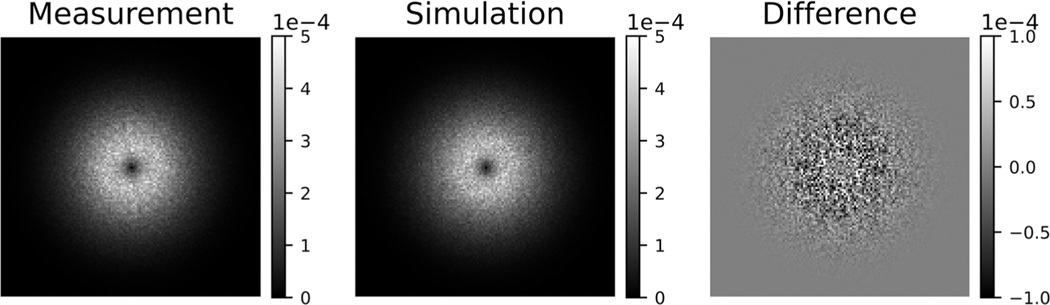
2D unstructured nNPS in the center region of measured (left) and simulated (middle) water phantom, and the difference between both nNPSs (right). Difference = simulation − measurement.

**FIGURE 12:**
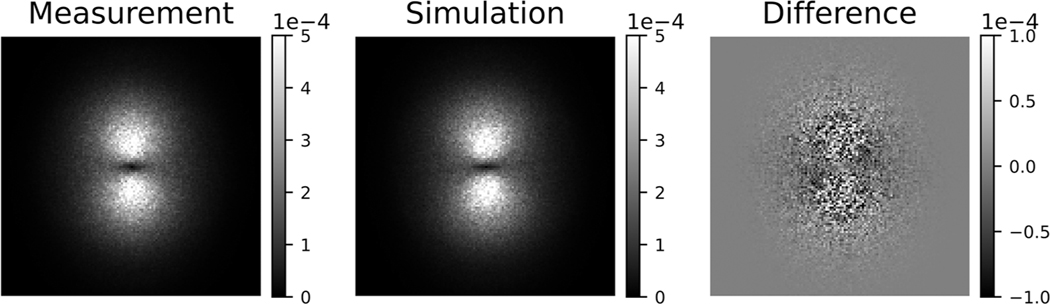
2D unstructured nNPS in the periphery region of measured (left) and simulated (middle) water phantom, and the difference between both nNPSs (right). Difference = simulation − measurement.

The ROIs used to validate the frequency content within a slice are also indicated in Figure 8, by the red and yellow squares. Please note that the ROIs of the measurements have a slight offset in vertical direction, this offset compensates for misalignment between the measured and simulated water phantom, to ensure that the same location of the phantom was analyzed. The 2D nNPS and 2D unstructured nNPS of the noise at the center (red square in Figure 8) within a slice of the measured and simulated phantom are shown in Figures 9 and 11, respectively. It can be seen that the 2D nNPS and 2D unstructured nNPS are isotropic in the center for both measurement and simulation. The difference images only show a small overestimation at lower frequencies (white dominant ring) and underestimation at slightly higher frequencies (black dominant ring). The 2D nNPS and 2D unstructured nNPS of the noise at the periphery (yellow square in Figure 8) of the measured and simulated phantom are shown in Figures 10 and 12, respectively. These results show that the nNPS is anisotropic at the periphery for both measurement and simulation, and that the degree of anisotropy in the simulated image is similar to that in the real one. The difference images only show a small overestimation in vertical direction (white dominant regions above and below the center) and underestimation in horizontal direction (black dominant regions left and right from the center).The only difference between the 2D normalized nNPSs and 2D unstructured normalized nNPSs is a small low frequency peak in the horizontal direction of the 2D nNPS of the periphery, which is not present in the 2D unstructured nNPS of the periphery. This minor peak is introduced by a small cupping artifact in the periphery of the measurement, see Figure 15.

**FIGURE 13:**
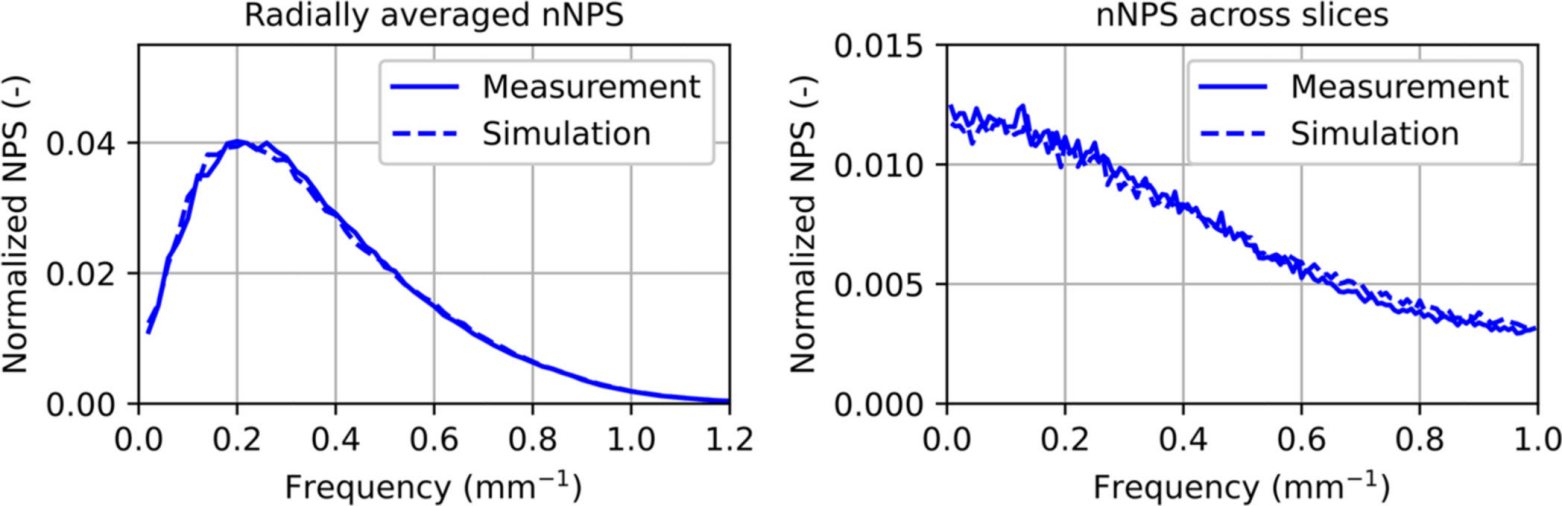
Radially averaged center nNPS (left) and nNPS across slices (right), for 135 kV and 140 mA.

Figure 13 shows the radially averaged nNPS and nNPS across slices in the center of both the measurement and simulation for the 135 kV and 140 mA case. The mean absolute errors of radially averaged nNPSs are summarized in Table 5, which shows that the maximum mean absolute error is 8.4% and the average mean absolute error is 3.9%. The mean absolute errors of the nNPSs in the slice direction are summarized in Table 5, and is on average 6.2% and maximum 8.8%. The plots of the other radially averaged nNPSs and nNPSs across slices are depicted in Appendix D.

**FIGURE 14:**
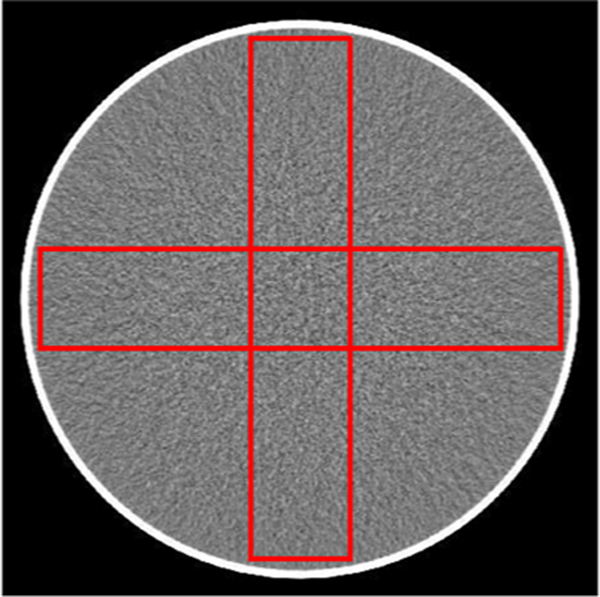
Averaged slices of simulated water phantom with indicated ROIs used for obtaining the line profiles.

**FIGURE 15:**
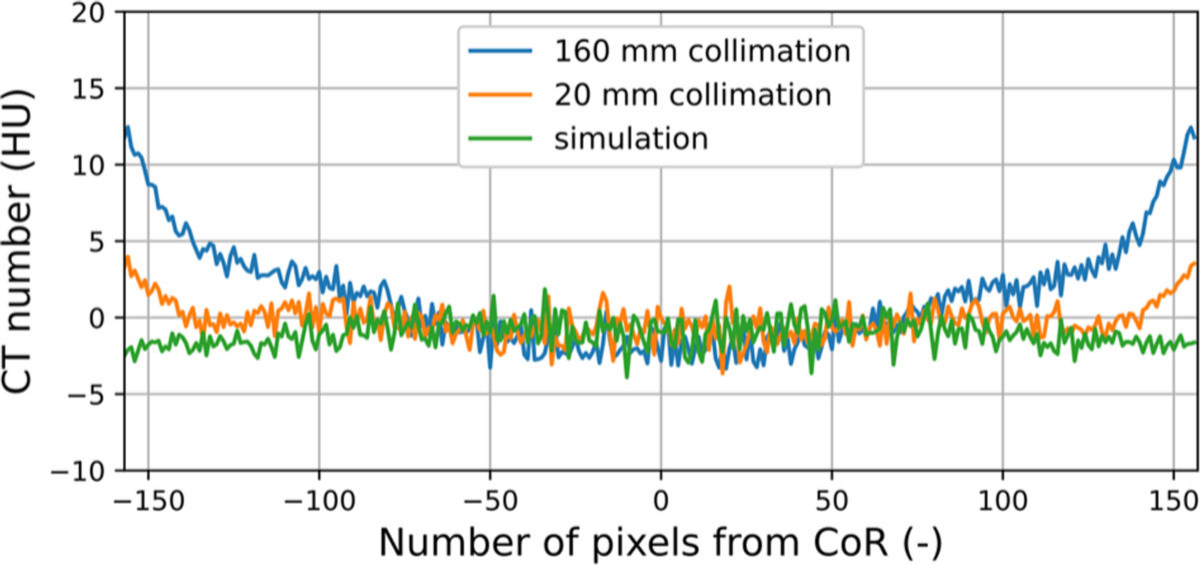
Line profiles of the measurement with 160 mm collimation, the measurement with 20 mm collimation, and the simulation.

Figure 14 indicates the region from where the line profiles of the water phantom for the scatter correction validation are obtained, with the resulting average line profiles shown in Figure 15. The line profiles show the effect of the scatter correction, resulting in a good match in HU values at the center of the phantom, but with a larger remaining error at the sides. As a result, it can be seen that the line profile of our simulation is also approximately flat, as expected, and has a maximum difference of 15 HU at the edge of the water phantom.

Tables 6 and 7 show the absolute relative errors between the simplified and the fully subsampled simulation of the frequency at 10% MTF for the simulated tungsten wire at 7, 14, and 21 cm from the CoR. The absolute relative errors for all simplifications are shown for the tangential (Table 6) and radial (Table 7) direction. In the last column of both Tables 6 and 7, the time and memory reduction factor are listed (the reduction factor is the same for time and memory, because the number of calculations that need to be performed scales linearly with the amount of memory in our application).

The differences in the resulting MTFs are, for most cases, subtle, except for the case of not subsampling the focal spot or angular projections. The detector subsampling seems to have very little effect in both directions.

Tables 6 and 7 also show the absolute relative errors in the frequency at 10% MTF between using the system MTF (as described in Section 2.7) and the fully subsampled focal spot simulation for the simulated tungsten wire at 7, 14, and 21 cm from the CoR. The results clearly show that using the system MTF improves the results when assuming the focal spot is a point source, since it reduces the error compared to the full simulation by approximately 30%−40%.The results also show that averaging each angular projection with the subsequent angular projection gives worse results than when simplifying to only using a single angular projection. Therefore, to have an error of 17% or less in the frequency at 10% MTF compared to the full simulation, all subsamples can be reduced to 1, except for the angular projections, which should still be 2. In this case the maximum error is 16.3% and the time and memory consumption could be reduced by a factor of 216.

**FIGURE 16:**
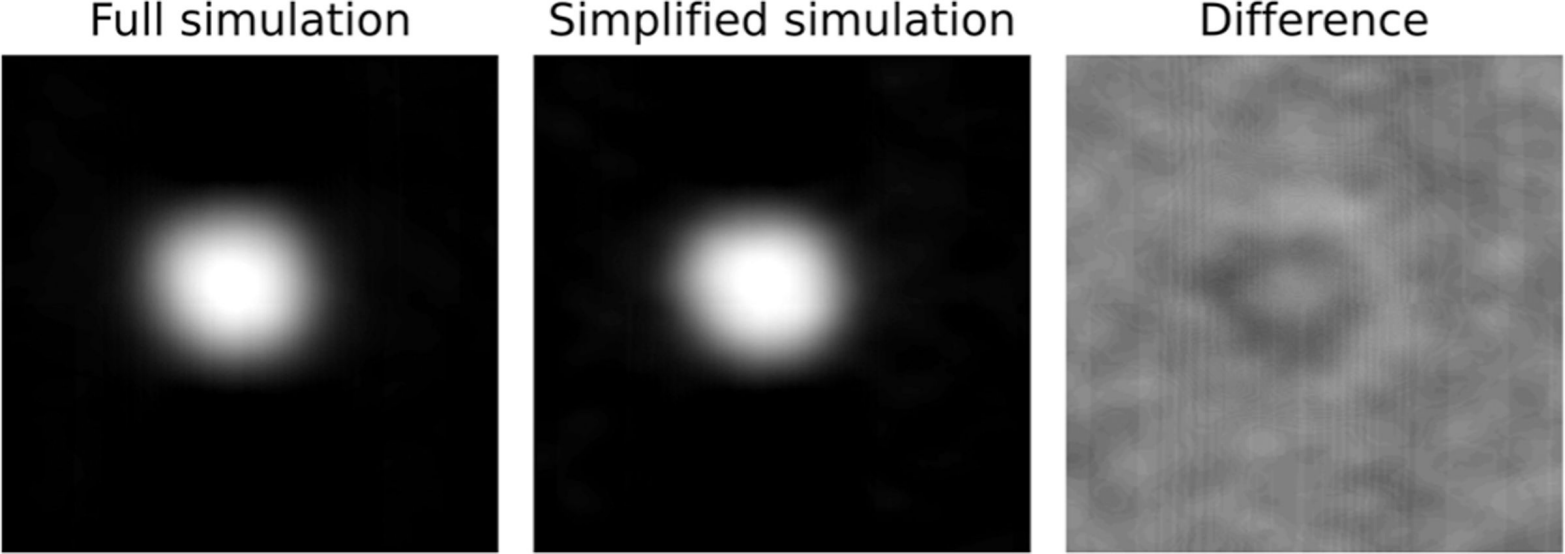
Noiseless simulations of a lesion at 14 cm from the CoR. (Left) Full simulation (WW: 100, WL: 85). (Middle) Simplified simulation, 1 source sample using the MTF measured in the CoR, 2 angular subsamples and 1 detector sample, time and memory consumption potentially reduced by a factor 216 (WW: 100, WL: 85). (Right) Difference between the two simulations (WW: 40, WL: 0).

**FIGURE 17:**
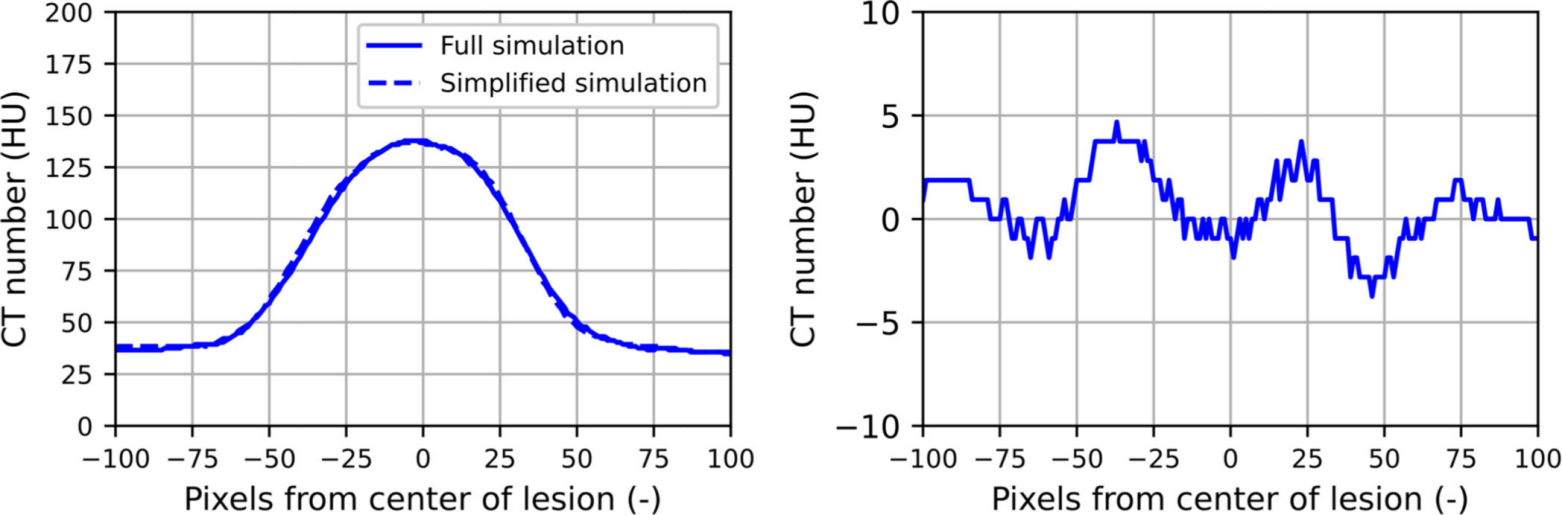
(Left) Line profile of the full and simplified simulation of the lesion in Figure 16. (Right) Difference between simplified simulation and full simulation (simplified simulation − full simulation).

The influence of these simplifications (resulting in 16.3% error) can be observed in Figure 16. Here a small lesion is imaged with full subsampling and simplified subsampling (1 source sample using the system MTF, 2 angular subsamples, and 1 detector sample) at 14 cm from the CoR, to show its influence. The difference image shows a minor ring but no other structural differences, indicating minimal difference between the two simulations after reconstruction. The same can be observed from the line profiles of these two reconstructed simulations plotted in Figure 17. However, for each application, the desired accuracy could differ, so the user should decide what is an acceptable error margin for their application.

### 3.1 | Computation time

All simulations were performed on a Linux system with 128 GB RAM,AMD Ryzen Threadripper 1950 × 16-core CPU, and a 48 GB Nvidia RX A600 GPU. The ray tracing and sinogram calculations were performed on the GPU. All image corruptions were performed on the CPU (due to the sinogram size). Generating all projections of 896 × 320 pixels for the MECT phantom, with a voxel array size of 50 × 1600 × 1600 voxels of the same voxel size as those used for validation and consisting of 15 different materials (used for CT number validation), took ∼40 h on this workstation. Please note that the time reduction factor reported with the simplification results is theoretical as some minor operations are independent of sinogram size and the calculations of an entire set of projections are done in batches due to memory constraints. As an example, by reducing the sub-sampling of the focal spot to 1, angular projections to 2, and detector pixels to 1, the simulation time for this same simulation results in ∼35 min.

## 4 | DISCUSSION

In this work a scanner-specific CT simulation was developed and validated, combining physics-based simulations for the position-dependent effects and the shift-invariant image corruption methods described by Saunders et al.^20^ for the detector effects. Therefore, minimizing the system information needed from the vendor and making it possible to perform scanner-specific CT simulations with only system-specific geometry information. In addition, the impact of simplifying the simulation process, both in terms of resulting realism and computer power requirements, was evaluated. To include all effects of the real CT system resulting in spatial resolution loss, the shift-variant impact of the finite focal spot size and of gantry rotation are modeled. The simulator is shown to generate images that match the characteristics of the real images to within an average of 3.4% and 3.3% in terms of spatial resolution within and across image slices, respectively. In terms of noise characteristics, these matched in terms of noise magnitude (standard deviation) and noise frequency content (nNPS) within and across slices to within 4.9%, 3.9%, and 6.2%, respectively. The effect of simulation simplification was assessed, and the results showed that for general applications most simplifications, except for the angular simplification, do not cause a major decrease in realism of the simulated image (maximum error in frequency at 10% MTF of 16.3%). Therefore, the time and computer power necessary could be reduced for many applications in which this level of realism is sufficient. This would aid studies that aim to use large virtual clinical trials, since it will become feasible to generate very extensive datasets within a reasonable time frame. Also, studies about processing or reconstruction algorithms could benefit as it becomes more feasible to cover large multi-dimensional parameter spaces for (first stage) testing, after which one could choose for more realistic simulations for refinement of solutions, if needed. However, the purpose of the study must be considered to make an informed decision on the level of realism that is desired.

During the development some assumptions had to be made. One of them being the assumption of stationary behavior of the detector MTF and NPS across the entire detector. The NPSs were only measured at the detector center, since the curvature of the detector is such that the normal direction of each element is pointing to the source. Only in the direction of the rows, where there is no curvature, the normal is not pointing directly to the source, however the maximum angle is 10° and therefore assumed to have a negligible effect. Also, the MTF was only measured in the detector row direction (since in the other direction the MTF is affected by the gantry rotation), then assuming rotational symmetry between all directions. In addition, the resolution characteristics are dominated by the focal spot size and finite exposure time during the angular motion of the CT gantry, justifying the assumption of stationary and symmetric MTF across the detector. Also, the residual scatter after correction was neglected. As shown, this simplification results in a CT number difference of approximately 15 HU at the edge of a 320 mm water phantom with the widest x-ray beam collimations. The QE of the detector is based on the theoretical energy absorption of the nominal detector active layer thickness. However, even with these assumptions, the validation results point to the appropriateness of the simulations in terms of spatial resolution, noise magnitude and frequency content, and, especially, their shift variance compared to a real clinical CT system.

The developed scanner-specific CT simulation may function as a tool to facilitate virtual clinical trials to test new reconstruction algorithms and post-processing algorithms. The simulator could additionally be used to also test new components like bowtie filters or flat filters, different detectors, or a different focal spot size, as well as new acquisition protocols.

## 5 | CONCLUSION

A scanner-specific CT simulation was developed, implemented, and validated. The validation of the performed simulations showed that it can generate images comparable to those obtained using a real clinical CT system. An analysis on simulation simplification also showed that for general applications, time and computer power can be spared without substantial loss of realism. The simulator can generate realistic scanner-specific CT images, which will aid the development of new reconstruction and post-processing algorithms by opening the possibility for virtual clinical trials.

## Supplementary Material

supp material

## Figures and Tables

**TABLE 1:** Computed tomography (CT) numbers corresponding to the regions indicated in the MECT phantom of Figure 4. The material with the maximum error in CT number is bold and underlined for each tube voltage. Note that the B in material 11 and 12 stands for blood with *ρ*= 1.03 g/cm^3^. The error is defined as simulation – measurement.

Material	100 kV		120 kV		135 kV	
	Measurement [HU]	Simulation [HU]	Measurement [HU]	Simulation [HU]	Measurement [HU]	Simulation [HU]

(1) Solid water	8.1	−0.6	3.9	−5.3	4.6	−4.0
(2) Adipose	−61.4	−74.7	−52.5	−69.7	−48.8	−66.2
(3) Brain	43.4	28.0	42.7	26.6	42.3	26.7
(4) 2 mg/mL I	63.6	54.1	51.6	38.6	48.3	35.4
(5) 5 mg/mL I	143.9	132.3	114.6	103.7	103.4	90.8
(6) 10 mg/mL I	267.4	263.5	206.2	203.8	178.0	177.5
(7) 15 mg/mL I	384.1	385.4	296.5	303.3	258.0	264.1
(8) Blood *ρ* = 1.03	54.8	41.8	45.5	38.8	44.8	41.3
(9) Blood *ρ* = 1.07	78.1	65.6	70.9	63.7	70.6	68.2
(10) Blood *ρ* = 1.1	106.0	97.4	104.7	97.3	106.3	100.3
(11) 2 mg/mL I + B	107.6	95.6	92.4	82.1	86.5	77.3
(12) 4 mg/mL I + B	149.6	140.2	127.0	119.6	117.8	111.2
(13) 50 mg/mL Ca	189.8	169.9	176.3	162.4	171.2	157.7
(14) 100 mg/mL Ca	**335.0**	**313.7**	**302.4**	**281.7**	**291.6**	**269.1**
(15) 300 mg/mL Ca	882.9	886.3	776.3	779.6	728.9	732.8
Mean absolute error [HU]	10.9		10.2		9.4	
Mean error [HU]	−10.3		−8.8		−8.1	

**TABLE 2:** Frequency at 10% MTF of the measured and simulated MTF, and the relative error of these frequencies in the different directions, positions, and focal spot sizes evaluated. Relative error = (simulation − measurement)/measurement * 100%.

Focal spot size	Direction	Position (cm)	Measured frequency (mm^−1^)	Simulated frequency (mm^−1^)	Relative error (%)

Large	Tangential	7	0.62	0.62	0.3
		14	0.57	0.57	1.2
		21	0.49	0.51	3.6

	Radial	7	0.62	0.65	3.8
		14	0.56	0.63	11.1
		21	0.37	0.38	2.6

Small	Tangential	7	0.72	0.70	−3.4
		14	0.64	0.63	−1.5
		21	0.55	0.55	0.8

	Radial	7	0.77	0.74	−3.3
		14	0.70	0.73	3.6
		21	0.41	0.39	−5.7

**TABLE 3:** Frequency at 10% SSP of the measured and simulated SSP, and the relative error of these frequencies for both focal spot sizes evaluated. Relative error = (simulation − measurement)/measurement * 100%.

Focal spot size	Measured frequency (mm^−1^)	Simulated frequency (mm^−1^)	Relative error (%)

Large	1.14	1.12	−1.7
Small	1.48	1.55	4.8

**TABLE 4:** Noise magnitude of measured and simulated water phantom of Figure 8. Relative error = (simulation − measurement)/measurement * 100%.

Tube voltage (kV)	Tube current (mA)	*σ* measurement (HU)	*σ* simulation (HU)	Relative error (%)

100	140	128.2	140.2	8.5
	400	66.8	68.8	2.9
120	140	83.8	89.0	5.8
	400	47.0	48.1	2.2
135	140	68.4	72.8	6.1
	400	39.0	40.4	3.6

**TABLE 5:** Difference in measured and simulated nNPS with a slice and across slices.

Tube voltage	100 kV		120 kV		135 kV	
Tube current	140 mA	400 mA	140 mA	400 mA	140 mA	400 mA

% Mean absolute difference within slice	2.2	2.2	2.4	4.9	3.1	8.4
% Mean absolute difference across slices	5.6	5.7	5.2	7.3	4.8	8.8

**TABLE 6:** Frequency at 10% MTF for all simulation simplifications and absolute relative error of this frequency compared to the frequency of the full simulation at 10% MTF, for tangential direction.

			Absolute relative error at 10% MTF			Time/memory reduction factor
Focal spot size	Subsampling simplification	Subsampling factor	7 cm	14 cm	21 cm	

Large	Focal spot	1	20.9	18.0	12.1	9
		2	7.7	8.5	4.1	2.25

	System MTF	1	12.5	11.9	7.6	9

	Angular	1	2.5	6.0	16.5	3
		2	0.4	0.7	1.8	1.5

	Projection averaging	1	15.2	14.5	15.3	3

	Detector	1	0.4	0.0	0.2	16
		2	0.1	0.0	0.0	4
		3	0.1	0.1	0.1	1.78

Small	Focal spot	1	6.9	6.6	3.8	9
		2	3.0	3.4	2.2	2.25

	System MTF	1	0.6	1.1	0.4	9

	Angular	1	2.5	10.3	20.1	3
		2	0.4	1.5	2.3	1.5

	Projection averaging	1	16.9	12.6	9.7	3

	Detector	1	0.2	0.0	0.4	16
		2	0.0	0.0	0.1	4
		3	0.0	0.1	0.1	1.78

**TABLE 7:** Frequency at 10% MTF for all simulation simplifications and absolute relative error of this frequency compared to the frequency of the full simulation at 10% MTF, for radial direction.

			Absolute relative error at 10% MTF			Time/memory reduction factor
Focal spot size	Subsampling simplification	Subsampling factor	7 cm	14 cm	21 cm	

Large	Focal spot	1	21.1	25.2	2.8	9
		2	11.2	10.4	1.7	2.25

	System MTF	1	12.7	16.3	3.4	9

	Angular	1	0.1	0.1	0.1	3
		2	0.1	0.0	0.0	1.5

	Projection averaging	1	0.6	1.0	0.4	3

	Detector	1	−0.6	2.1	0.1	16
		2	0.2	0.3	0.1	4
		3	0.3	0.1	0.1	1.78

Small	Focal spot	1	5.5	7.7	0.8	9
		2	3.7	3.8	0.5	2.25

	System MTF	1	1.8	0.0	1.3	9

	Angular	1	0.0	0.1	0.0	3
		2	0.1	0.0	0.0	1.5

	Projection averaging	1	1.1	1.3	0.1	3

	Detector	1	1.2	1.3	0.2	16
		2	0.1	0.2	0.0	4
		3	0.0	0.1	0.0	1.78
