# Supplementary material for: Development, validation, and simplification of a scanner-specific CT simulator
Source: Med Phys. Author manuscript; Available in PMC 2024 Mar 14. (PMC10904672; doi:10.1002/mp.16679)
Supplement: supp material [file NIHMS1939930-supplement-supp_material.pdf]

APPENDIX A – General overview pipeline

The general simulation pipeline in Fig. A1 gives an overview of all information or measurements required per step, so the same method can be applied for simulating other CT systems.

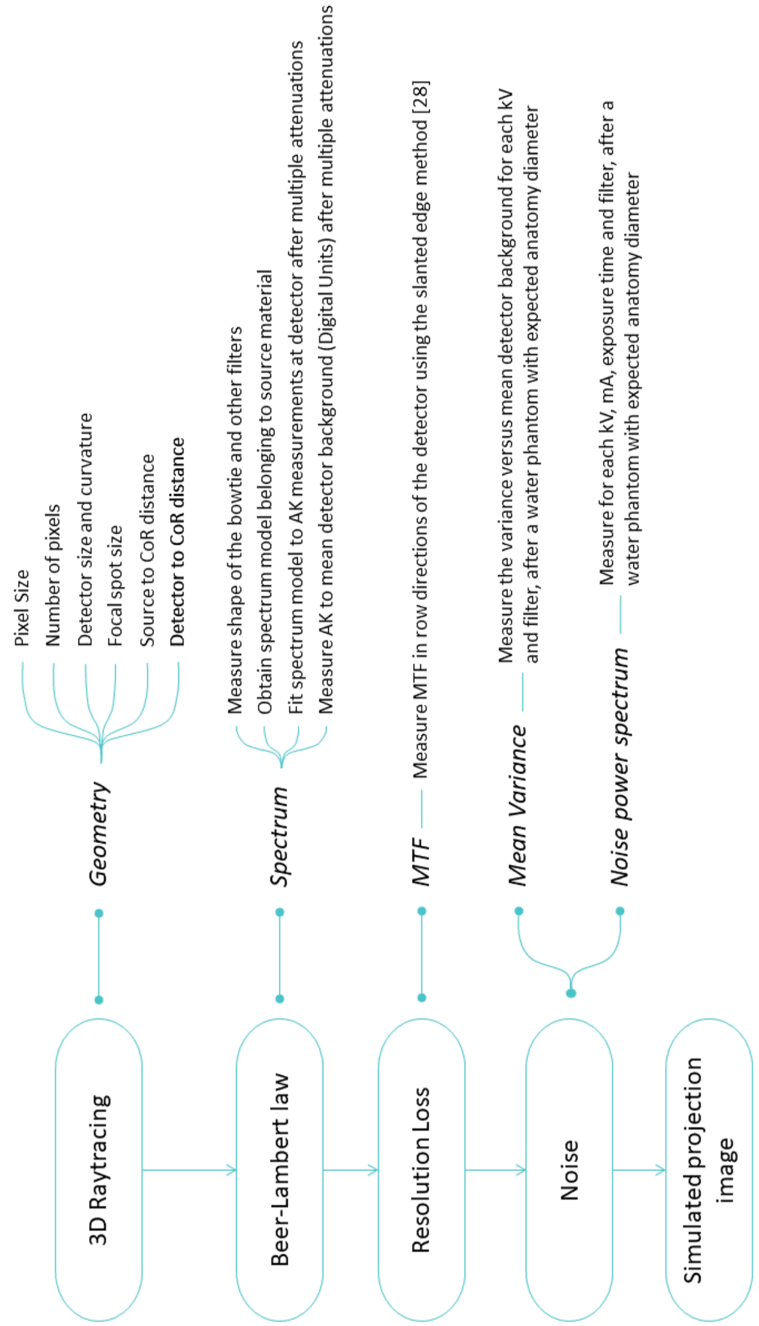

Fig. A1: General pipeline presenting all required information or measurements per simulation step.

APPENDIX B - Modulation Transfer Functions

A tungsten edge (TX5, IBA Dosimetry, Schwarzenbruck, Germany) was used in combination with the slanted edge method[28] to measure the edge spread function in the row direction of the detector. Since this direction does not experience resolution loss due to rotation and obtaining linear domain data with the gantry remaining static was not possible. To determine the detector MTF, the tungsten edge was placed as close as possible to the detector entrance surface (8 cm away), to minimize the focal spot size effect.

The function used to fit the MTF is based on the Lorentzian fit of Siewerdsen et al.[29], defined as follows:

$$MTF(f) = \frac{1}{1 + a * f + b * f^2 + c * f^3 + d * f^4 + e * f^5} \quad (6)$$

where f is the spatial frequency and a, b, c, d, and e are the optimizable parameters of the function.

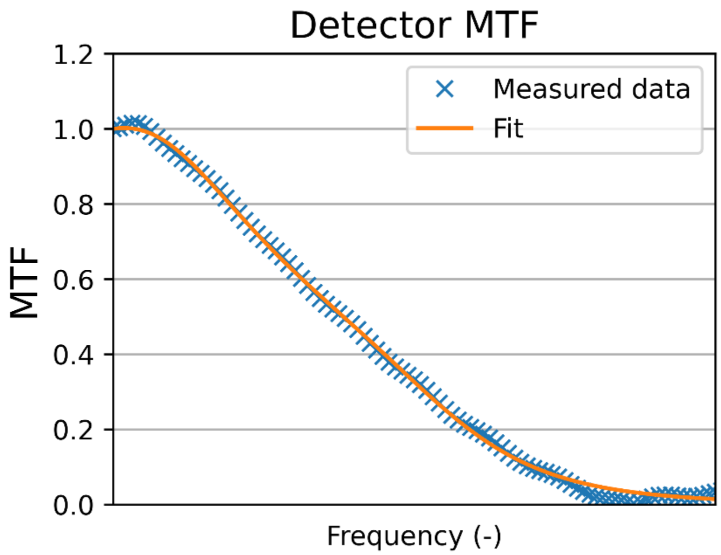

Fig. B1: Measured data and fit of the detector MTF.

The data points and fitted MTF are shown in Fig. B1. The R^2 of the fit is listed in Table B1. Please note that the values of the x-axis are not displayed, and the fitted parameters are not reported, since these are confidential.

The same measurements have been performed at the center of rotation (CoR) to determine the system MTF for both focal spots present in the system. The data points and fitted MTF curves are shown in Fig. B2. The resulting R^2 of the fits are listed in Table B1 for both focal spot sizes. Please note that the values of the x-axes are not displayed, and the fitted parameters are not reported, since these are confidential.

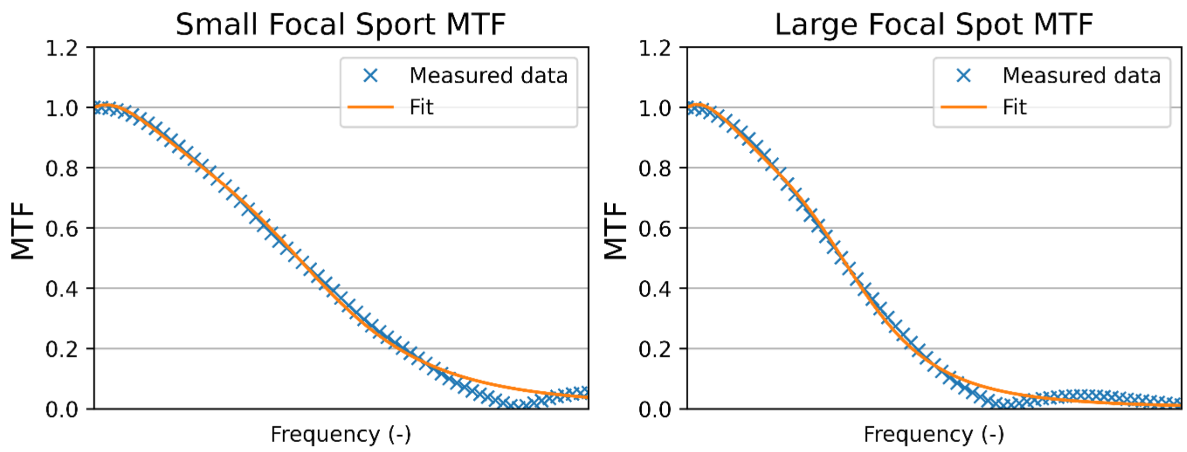

Fig. B2: Measured data and fit of the system MTF of both focal spots present in the system.

Table B1: R^2 values for fits.

	R^2
**Detector MTF**	0.998
**System MTF (small focal spot)**	0.996
**System MTF (large focal spot)**	0.998

APPENDIX C – Subsampling Experiments

The number of subsamples required for the focal spot (LxL), detector elements (MxM), and angular projections (K) were determined experimentally. Please note that the values of the x-axes are not displayed, since these are confidential.

To determine the number of focal spot subsamples, a tungsten edge (TX5, IBA Dosimetry, Schwarzenbruck, Germany) was placed at the Center of Rotation (CoR) and imaged using the large focal spot of the system. This tungsten edge was also simulated at the CoR. The MTF in the sinogram was determined for both directions and for both measurement and simulation. Experimental validation showed that the optimal number of focal spot subsamples per direction is $L = 3$, see Fig. C1 and Fig. C2.

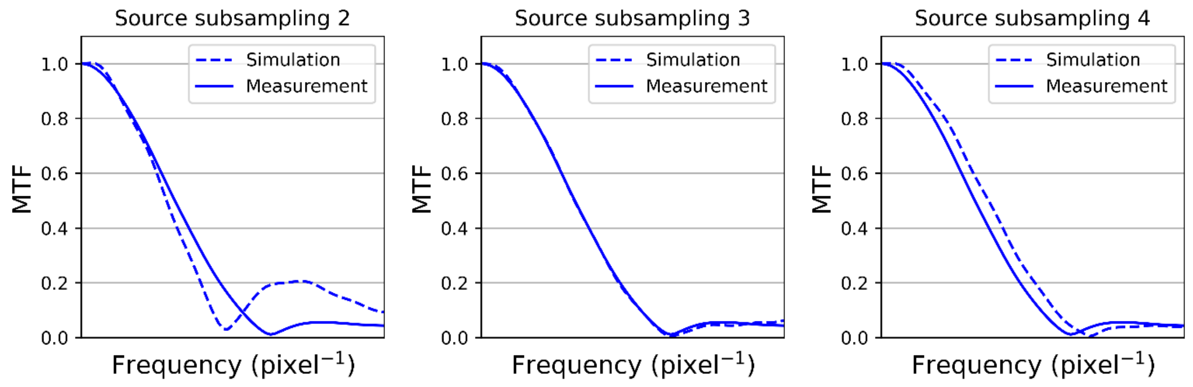

Fig. C1: MTF in the row direction of the detector with, from left to right 2, 3, and 4 subsamples in each direction of the focal spot.

The number of subsamples for the detector elements was determined by placing the tungsten edge as close as possible to the detector entrance (8 cm away), to minimize the focal spot effect, and imaging it with the small focal spot present in the system. The MTF in the sinogram was determined only for the direction across detector rows since the other direction has the angular rotation effect. Experiments showed that the optimal number of detector subsamples per direction is $M = 4$, see Fig. C3, since lower subsamples result in the minimum of the MTF being at a too low frequency.

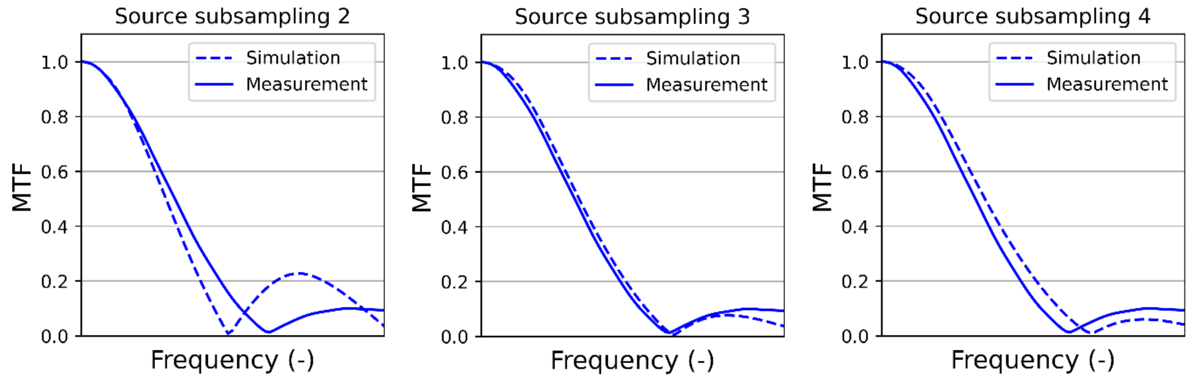

Fig. C2: MTF in the column direction of the detector with, from left to right 2, 3, and 4 subsamples in each direction of the focal spot.

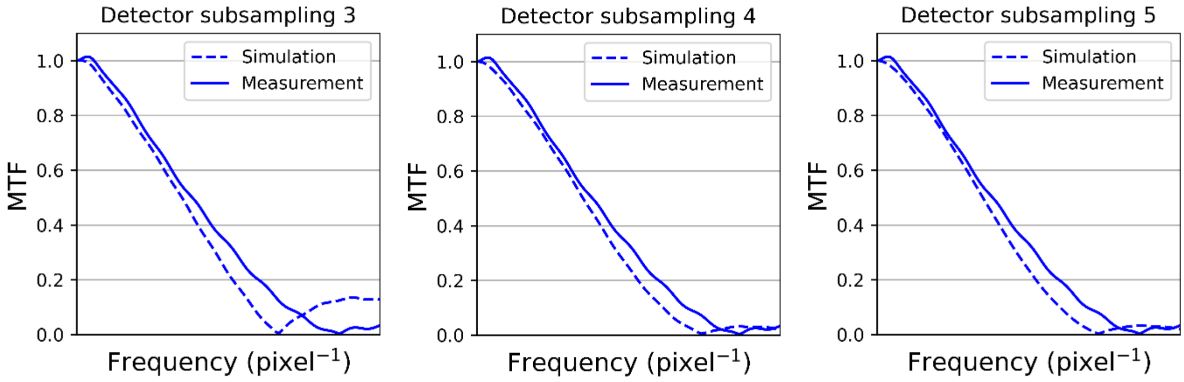

Fig. C3: MTF in the row direction of the detector with, from left to right 3, 4, and 5 subsamples in each direction of the detector pixels.

To determine the number of angular projections subsamples the tungsten edge was again placed as close as possible to the detector entrance (8 cm away) and imaged with the small focal spot. The MTF in the sinogram was determined only for the direction across detector columns, as this is the only direction in which the resolution loss is influenced by the angular rotation. Experimental validation showed that the optimal number of angular projection subsamples is $K = 3$, see Fig. C4, since higher subsampling does not show significant improvement.

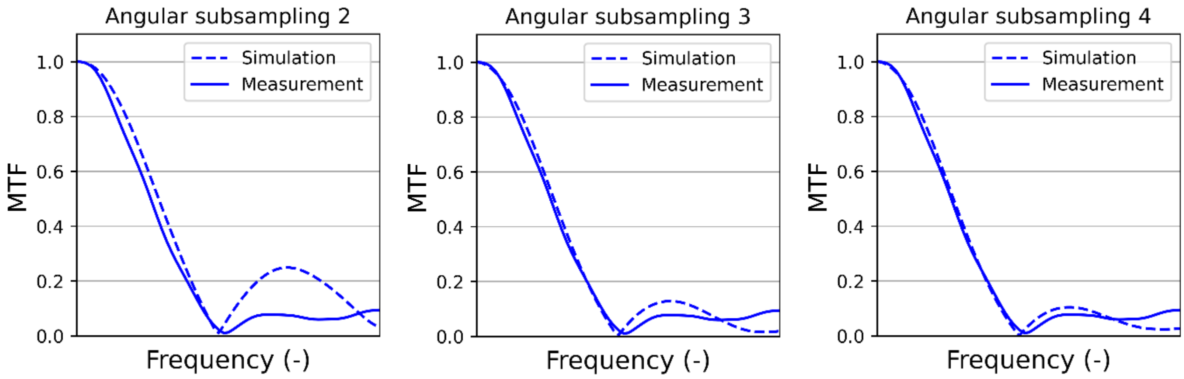

Fig. C4: MTF in the column direction of the detector with, from left to right 2, 3, and 4 subsamples for each angular projection.

APPENDIX D – Noise Power Spectra

The radially averaged nNPS and nNPS across slices in the center of both the measured and simulated water phantom of Fig. 8 of the manuscript, at tube voltage levels of 100 kV, 120 kV, and 135 kV and tube current levels of 140 mA and 400 mA are shown in Fig. D1-D5. Please note that the 135 kV and 140 mA case is not shown, since it is already shown in Fig. 13 of the manuscript.

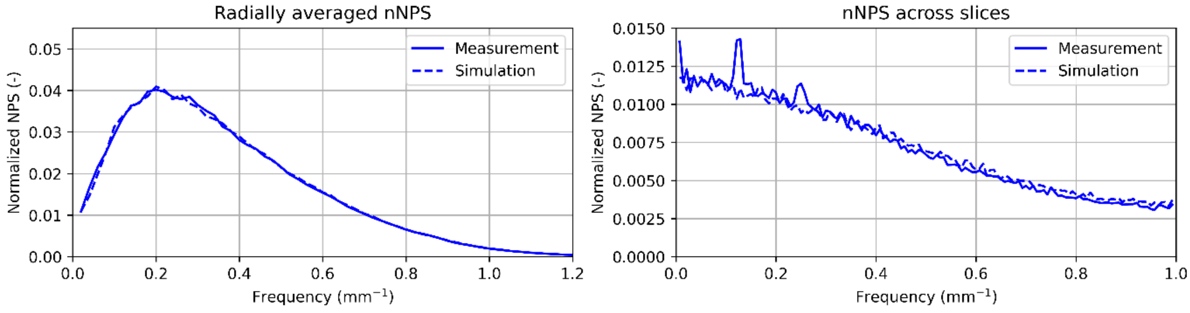

Fig. D1: Radially averaged center nNPS (left) and nNPS across slices (right), for 100 kV and 140 mA.

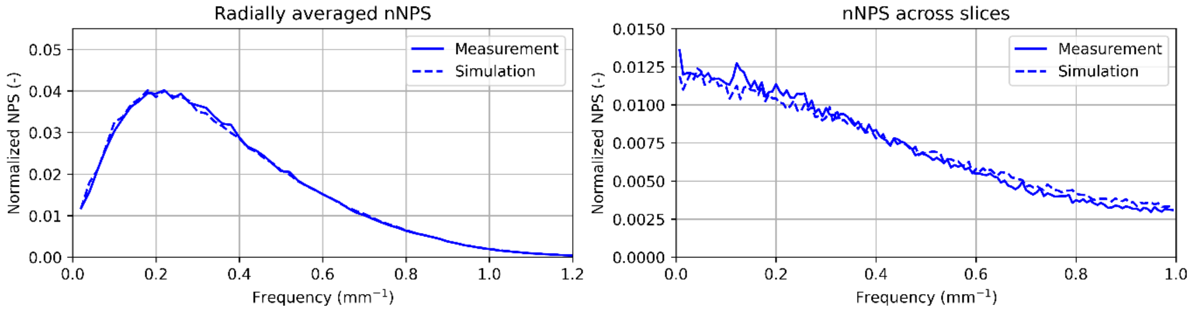

Fig. D2: Radially averaged center nNPS (left) and nNPS across slices (right), for 100 kV and 400 mA.

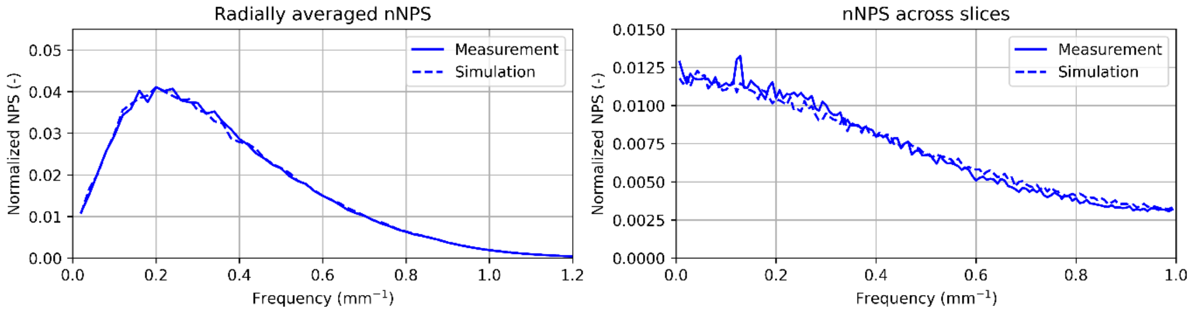

Fig. D3: Radially averaged center nNPS (left) and nNPS across slices (right), for 120 kV and 140 mA.

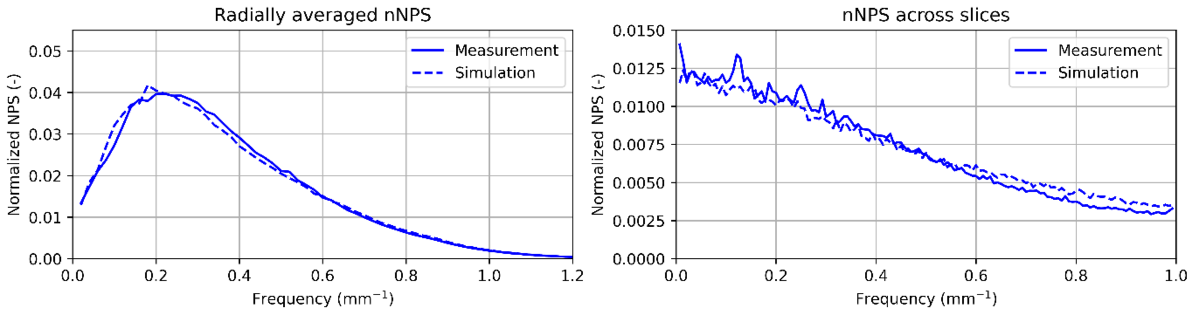

Fig. D4: Radially averaged center nNPS (left) and nNPS across slices (right), for 120 kV and 400 mA.

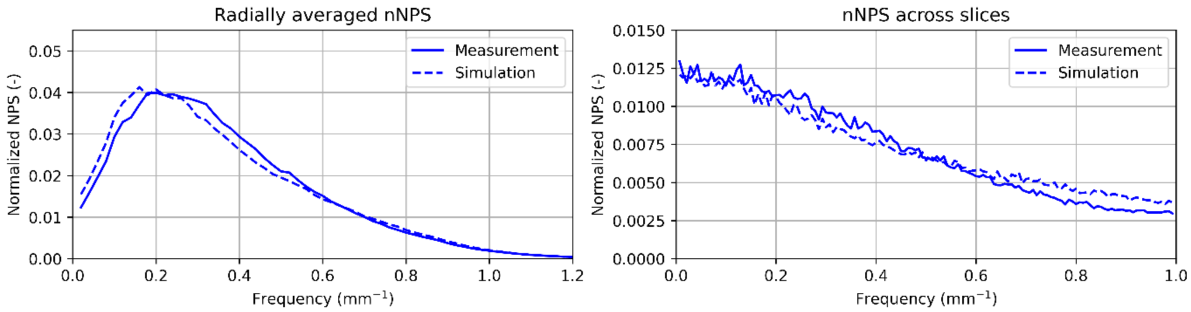

Fig. D5: Radially averaged center nNPS (left) and nNPS across slices (right), for 135 kV and 400 mA.
